# Mutational Signatures of De-Differentiation in Functional Non-Coding Regions of Melanoma Genomes

**DOI:** 10.1371/journal.pgen.1002871

**Published:** 2012-08-09

**Authors:** Stephen C. J. Parker, Jared Gartner, Isabel Cardenas-Navia, Xiaomu Wei, Hatice Ozel Abaan, Subramanian S. Ajay, Nancy F. Hansen, Lingyun Song, Umesh K. Bhanot, J. Keith Killian, Yevgeniy Gindin, Robert L. Walker, Paul S. Meltzer, James C. Mullikin, Terrence S. Furey, Gregory E. Crawford, Steven A. Rosenberg, Yardena Samuels, Elliott H. Margulies

**Affiliations:** 1 National Human Genome Research Institute, National Institutes of Health, Bethesda, Maryland, United States of America; 2 Institute for Genome Sciences and Policy, Duke University, Durham, North Carolina, United States of America; 3 Department of Pathology, Memorial Sloan-Kettering Cancer Center, New York, New York, United States of America; 4 National Cancer Institute, National Institutes of Health, Bethesda, Maryland, United States of America; 5 Graduate Program in Bioinformatics, Boston University, Boston, Massachusetts, United States of America; 6 Department of Genetics, Department of Biology, Lineberger Comprehensive Cancer Center, and Carolina Center for Genome Sciences, The University of North Carolina at Chapel Hill, Chapel Hill, North Carolina, United States of America; University of Washington, United States of America

## Abstract

Much emphasis has been placed on the identification, functional characterization, and therapeutic potential of somatic variants in tumor genomes. However, the majority of somatic variants lie outside coding regions and their role in cancer progression remains to be determined. In order to establish a system to test the functional importance of non-coding somatic variants in cancer, we created a low-passage cell culture of a metastatic melanoma tumor sample. As a foundation for interpreting functional assays, we performed whole-genome sequencing and analysis of this cell culture, the metastatic tumor from which it was derived, and the patient-matched normal genomes. When comparing somatic mutations identified in the cell culture and tissue genomes, we observe concordance at the majority of single nucleotide variants, whereas copy number changes are more variable. To understand the functional impact of non-coding somatic variation, we leveraged functional data generated by the ENCODE Project Consortium. We analyzed regulatory regions derived from multiple different cell types and found that melanocyte-specific regions are among the most depleted for somatic mutation accumulation. Significant depletion in other cell types suggests the metastatic melanoma cells de-differentiated to a more basal regulatory state. Experimental identification of genome-wide regulatory sites in two different melanoma samples supports this observation. Together, these results show that mutation accumulation in metastatic melanoma is nonrandom across the genome and that a de-differentiated regulatory architecture is common among different samples. Our findings enable identification of the underlying genetic components of melanoma and define the differences between a tissue-derived tumor sample and the cell culture created from it. Such information helps establish a broader mechanistic understanding of the linkage between non-coding genomic variations and the cellular evolution of cancer.

## Author Summary

Here we investigate the relationship between somatic variants and non-coding regulatory regions. To do this, we develop a new algorithm for identifying single nucleotide somatic variants in whole-genome sequencing data and apply it to a metastatic melanoma sample and a cell culture derived from this sample. Our results show that the two genomes are similar at the level of single nucleotide changes and more variable at larger copy number changes. We further observe that patterns of somatic mutation accumulation in non-coding regulatory regions suggests that the metastatic melanoma cells de-differentiated into a more basal regulatory state. That is, by simply looking at mutation accumulation across cell-type-specific non-coding functional regions, one can clearly see patterns that are indicative of cell state de-differentiation. Results from genome-wide functional regulatory region experimental mapping support this observation.

## Introduction

Sporadic cancer is mainly caused by the progressive accumulation of genomic mutations. Therefore, a mechanistic understanding of cancer requires a comprehensive catalog of all somatic variants in a tumor genome. Although the majority of somatic variants occur in non-coding regions of the genome, most studies have focused on interpreting genic mutations [1], even when whole-genome data was generated [1]–[6]. As a consequence, it is unclear if and how non-coding variants might contribute to cancer progression. To comprehensively study functional consequences of somatic variants, one needs cell cultures made from the tumor. First, though, one needs to know how representative the cell culture is compared to the original cancerous tissue. Here we characterize these differences and use comparative and functional genomics methods to assess how mutations are distributed within melanoma genomes.

**Figure 1:**
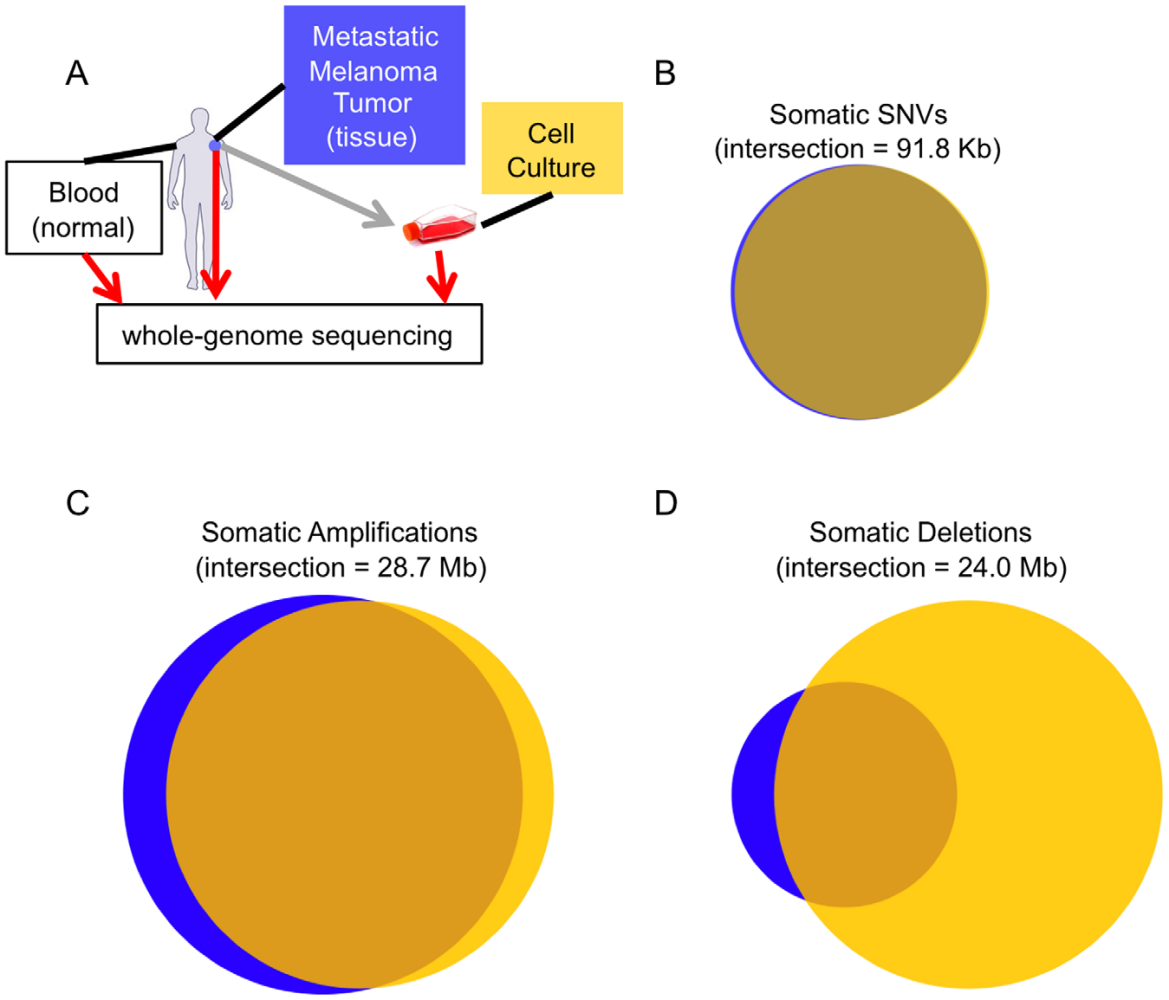
Melanoma tissue and cell culture similarities. (A) The experimental design of our study. Concordance between the somatic calls in the tissue (blue) and cell culture (yellow) for SSNVs (B), CNV amplifications (C), and CNV deletions (D). The two samples are highly concordant at the SNV level, but more different at the CNV level.

We used a combination of data produced by the Illumina GAIIx and HiSeq2000 platforms to generate over 5.4 billion 100 bp reads representing three different high-coverage genomes (Figure 1A and S1) from the same 33 year old untreated male: two genomes represent a cutaneous melanoma sample, one of a laser capture microdissected metastatic tumor from the shoulder (primary tumor is of unknown origin), and the other from a low-passage cell-culture derived from that tumor. We also generated a matched “normal” genome from a blood sample. Using our single nucleotide genotype calling methodology [7], we were able to make confident genotype calls at 92.9%, 84.5%, and 95.6% of the tissue, cell culture, and normal genomes, respectively.

## Results/Discussion

### Analysis of detected variants

To accurately and comprehensively identify novel somatic single nucleotide variants (SSNVs) in the cell culture and tissue genomes we developed a new computational algorithm, which was validated and shown to have high sensitivity and specificity (see Materials and Methods). Utilizing published algorithms [8]–[10], we were also able to identify somatic copy number changes and chromosomal rearrangements. Comparing the somatic alterations identified in the tissue and cell culture genomes reveals their extent of relatedness (Figure 1 and S2).

In total, we identified 105,460 SSNVs in the tissue and 122,837 in the cell culture that were not present in the patient's non-tumor DNA. This number of somatic mutations is substantially higher than other published whole-genome cancer studies [1]–[6]. If we examine genomic regions that have sufficient coverage to make a reliable call in both samples (81.1% of the genome), 95.2% of the sites are common (2.9% and 1.9% are unique to the tissue and cell culture, respectively). The two melanoma samples are less concordant at the level of copy number variations (CNVs) relative to the normal genome (Figure 1C and 1D). In total, 118 Mb of the cell culture has somatic CNVs whereas only 63 Mb of the tissue does. In support of these results, we found that aCGH CNV calls were highly concordant with our whole-genome sequencing-based calls (Figure S3). One striking difference in the cell culture genome is that it includes a near-complete loss of one copy of chromosome 14 (Figure S2 and S4). The additional CNVs in the cell culture genome may result from the low-passage culturing process. This is a known phenomenon that has been previously documented in higher-passage hESC cell cultures [11] and a xenograft of a primary tissue cancer sample [6]. As such, our CNV results are consistent with other reports and extend these findings to lower-passage tumor cell cultures. Because non-normal CNV regions can influence SSNV calls, we recalculated concordance at non-CNV regions. Focusing on these areas, there are 91,823 SSNVs in the union of both samples, and 96.1% are shared (2.0% and 1.9% are unique to the tissue and cell culture, respectively) (Figure 1B). The SSNV mutational spectrum is reflective of UV damage, even for cell culture and tissue-specific calls (Figure S5). We additionally made somatic insertion and deletion (indel) calls (see Materials and Methods), and found that after CNV filtering there are 269 somatic indels shared between the tissue and cell culture, while the tissue has 127 unique indels and the cell culture has 160 (Figure S6). This lower level of concordance, relative to SSNVs, between calls is not surprising, as previous studies show that indel calling is more difficult with short reads [12]. Together, these results provide a high-resolution picture of the differences between a metastatic tissue sample and the cell culture derived from it.

We next compared mutations from another melanoma whole-genome study by applying our computational SSNV detection method to sequence data from metastatic melanoma (colo-829) and matched normal (colo-829BL) cell lines [1]. We identify more SSNVs than originally reported, and the bulk of our calls are concordant with those (Figure S7). Importantly, we identify 448 of the 454 (98.7%) Sanger-validated and 40 of the 43 (93%) COSMIC calls in the colo-829 genome. Variant calls that are specific to our algorithm are enriched for the characteristic melanoma UV mutational signature (Figure S8). We observe 100% concordance with Sanger sequencing-based cross-validation of novel SSNV calls at 181 positions in the cell culture genome (see Materials and Methods), which suggests that our SSNV detection algorithm has a low false positive rate.

Additionally, we randomly selected 96 cell culture-specific and 96 tissue-specific SSNVs for PCR amplification and Sanger sequencing. Of the successful PCR and Sanger sequencing reactions, we observe 97.7% concordance and 98.7% concordance at tissue-specific and cell culture-specific positions, respectively. Together, these results suggest our SSNV detection algorithm is both highly sensitive and specific.

### Identification of commonly mutated genes in melanoma

Comparison of the colo-829 SSNVs to those from our melanoma sample shows commonly mutated genes, some of which are associated with melanoma pathogenesis (Figure S9). For example, missense mutations (D261N and H533Y) were identified in *ADAM29*, which encodes a member of the A Disintegrin And Metalloproteinase (ADAMs) family which are membrane anchored glycoproteins with several biological functions encompassing cell adhesion, cell fusion and signaling [1]. Importantly, we recently reported that a systematic mutational analysis of all members of the ADAM family of membrane-bound metalloproteases showed that *ADAM29* is often mutated in melanoma [13]. Functional analyses have indicated that *ADAM29* mutations affect adhesion of melanoma cells to specific extracellular matrix proteins, suggesting that mutated *ADAM* genes play a role in melanoma tumorigenesis [13].

This study also identified a missense mutation (R175C) in *PTK2B*, which encodes the non-receptor protein tyrosine kinase PTK2B, also known as PYK2 or FAK2, a focal adhesion protein that shares structural similarity with its paralog focal adhesion kinase 1 (FAK1). PTK2B has been previously linked to metastasis via RhoC-dependent activation of FAK1, MAPK, and Akt [14]. As we previously reported a high prevalence of somatic mutations in *PTK2B* in metastatic melanoma [15], these studies suggest that *PTK2B* may be a melanoma cancer gene and that further studies are required to more fully characterize the functional role of its mutations in melanoma. For additional genic annotations, we include a supplementary file that outlines all coding mutations discovered in this study (Table S1).

### Nonrandom mutation accumulation across the genome

Because metastatic tumor formation involves successive iterations of mutation, followed by selection and clonal expansion, the resulting cell population has undergone an evolutionary process commonly referred to as clonal evolution [16], [17]. When measuring the similarity of sequences across many species, the genome has clear signatures of intense selective pressure [18]–[23]. Some regions reject mutations more than expected. To determine if the selective forces operating on a metastatic cell over the span of cancer development are similar to those operating across species over millions of years, we compared somatic mutation accumulation in melanoma to evolutionary constraint.

**Figure 2:**
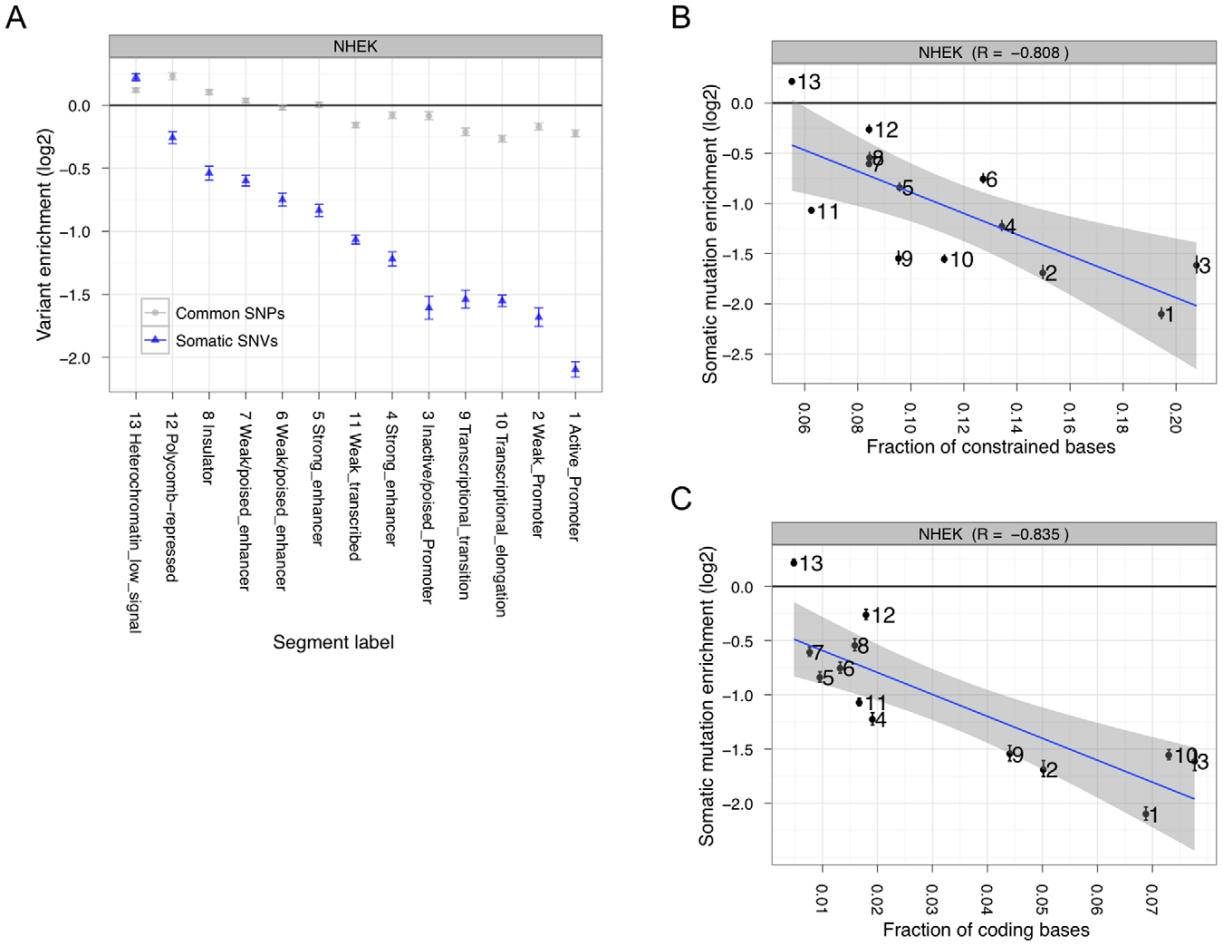
Somatic mutation accumulation is non-random across the genome. (A) Somatic (blue) and common (gray) variants have different levels of enrichment or depletion depending on which chromatin segmentation they occur in. Somatic mutation accumulation is highly anti-correlated with evolutionary constraint (B) and coding fraction (C).

For this analysis, we combined the SSNVs from our tissue sample with those we identified in the colo-829 cell line. This resulted in 141,655 unique SSNVs, of which 99.3% are non-coding. To determine if these mutations are uniformly distributed throughout the genome, we first measured mutation accumulation in functionally different regions identified by chromatin-based chromosomal segmentations [24] (Figure 2A). Such segmentations currently exist for nine different cell types (Figure S10), and we chose NHEK cells as our primary focus since these appear most similar to melanoma cells out of all nine cell types (see below). The enrichment results are consistent when the samples are analyzed independently (Figure S11). There is a clear anti-correlation with evolutionary constraint (Figure 2B and S12). However, there is also a strong anti-correlation with mutation accumulation and coding regions (Figure 2C and S13), which is expected due to transcription-coupled repair (TCR). Of note, the heterochromatin low signal regions (state 13 in the chromosomal segmentations) accumulate mutations roughly equal to random expectation (Figure S14), indicating that they may be suitable targets for estimating the background passenger somatic mutation rate (which for this tumor we calculate as about 42 SSNVs per megabase).

The above results indicate that somatic mutations do not occur uniformly across the genome. To eliminate the mutation suppression bias related to TCR in known genic areas, we specifically focused on regions of the genome less likely to be transcribed— windows that do not overlap and are greater than 10 Kb from annotated genes or transcription start sites (TSSs). We performed a multiple regression on mutation accumulation in bins of these regions using evolutionary constraint, GC content, and fraction of transcribed bases, which we obtained from a separate melanoma RNA-seq study [25]. Adding the additional variables removes the correlation with evolutionary constraint. Unsurprisingly, the fraction of non-coding bases transcribed (one of the variables in the above-mentioned regression analysis) is almost perfectly anti-correlated with enrichment for mutation accumulation (Spearman's R = −0.97789; P<2.2e-16). These results suggest that TCR is a mechanism associated with preventing mutation accumulation in non-coding regulatory elements.

### Functional mutation signatures in non-coding regulatory regions

**Figure 3:**
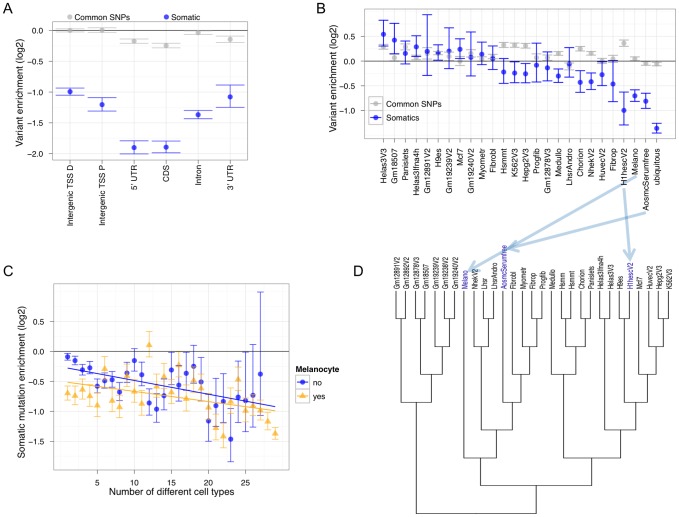
Non-coding Melanocyte DHSs are dis-enriched for accumulating melanoma somatic mutations. (A) Genic partitioning of melanocyte DHSs such that every DHS occurs in a single category shows that most categories are depleted for mutation accumulation (TSS P = Transcription Start Site Proximal [within 5 Kb]; TSS D = Transcription Start Site Distal [greater than 5 Kb]). Common SNPs are based on 1000 Genomes calls that have at least 5% minor allele frequency (MAF). (B) Intergenic TSS-distal cell-type-specific and ubiquitous DHSs show different levels of enrichment or depletion. (C) Enrichment or depletion at cell-type combinations of intergenic TSS-distal melanocyte and non-melanocyte DHSs. For these analyses, the set of regions representing any data point must have overlapped at least 10 somatic variants to be considered. The horizontal black line at zero represents no enrichment. The GSC method was used to measure enrichment. Error bars represent one standard deviation from the mean of the null distribution. (D) A hierarchical tree based on DHS Euclidean distance among 29 different cell states. Note the positioning of melanocytes “Melano” relative to aortic smooth muscle cells “AosmcSerumfree” and human embryonic stem cells “H1hesc”, which are among the most depleted for somatic mutation accumulation (Figure 3B).

We next sought to examine the distribution of somatic mutations across experimentally-derived functional non-coding regions. To do this, we compared our SSNV collection to broad classes of active regulatory elements identified by the DNaseI hypersensitive site (DHS) assay [26]–[30]. This experiment was performed genome-wide on melanocytes—the precursor cell type to melanoma—as part of the ENCODE Project Consortium [31]. We hierarchically partitioned melanocyte DHSs based on genic landmarks and calculated somatic mutation enrichment (Figure 3A). All DHS categories except for 3′ UTRs are significantly less enriched than random expectation (horizontal line at 0) and compared to common SNPs from the 1000 genomes consortium (grey points). 5′ UTRs are the most depleted. These results are consistent with the observed increase in mutation accumulation along the length of genes (Figure S15) and are reproducible when the samples are analyzed independently (Figure S16). Despite their distant location from known transcribed regions, intergenic TSS-distal DHSs are also significantly depleted for accumulating mutations. To avoid confounding from transcription-coupled repair (described above), we subsequently focus on intergenic TSS-distal DHSs. We performed single linkage clustering of DHSs from 29 different cell states (cell types and conditions) identified by the ENCODE Project Consortium [31] to identify sites that are cell-type-specific, present in a combination of cell types, or ubiquitously present. Out of all cell-type-specific DHSs, the most depleted for mutation accumulation are those specific to melanocytes, aortic smooth muscle cells (ASMCs) and H1 embryonic stem cells (ESCs). Ubiquitously present DHSs are even more depleted. We next calculated the mutational load on all melanocyte DHSs by measuring mutation accumulation when these regulatory regions are active in all possible cellular contexts/combinations. Unsurprisingly, mutation enrichment decreases as the melanocyte DHS is active in more cell types (Figure 3C; yellow line). As a control for this experiment we examined all combinations of non-melanocyte DHSs and found a similar trend (Figure 3C; blue line), although not as depleted as the melanocyte DHSs. These results indicate that regulatory regions are preferentially repaired in metastatic melanoma and that this occurs in a cell-type specific manner.

To further understand the relationship between cell type regulatory architecture and somatic mutation enrichment in metastatic melanoma, we clustered all 29 cell types based on their regulatory element signatures (Figure 3D). Note the relationship between melanocytes and the other two cell types where cell-type-specific DHS mutations are highly depleted (ASMCs and ESCs). ASMCs are derived from the same embryological layer—neural crest—as melanocytes, and ESCs are an undifferentiated pluripotent cell type. Brain cell (medulloblastoma)-specific DHSs, which are also neural crest derived, show significant depletion as well. The topology of the tree and the significant depletion for somatic mutation accumulation in regulatory regions specific to neural crest-derived and ESC cell types suggests that the metastatic melanoma cell utilized these regulatory programs. These results imply that the regulatory architecture of the metastatic melanoma cell de-differentiated to a more basal cellular program that is visible in the pattern of mutations covering cell-type-specific regulatory regions. In support of this hypothesis, a recent study found that human melanoma-initiating cells express a neural crest stem cell marker [32].

**Figure 4:**
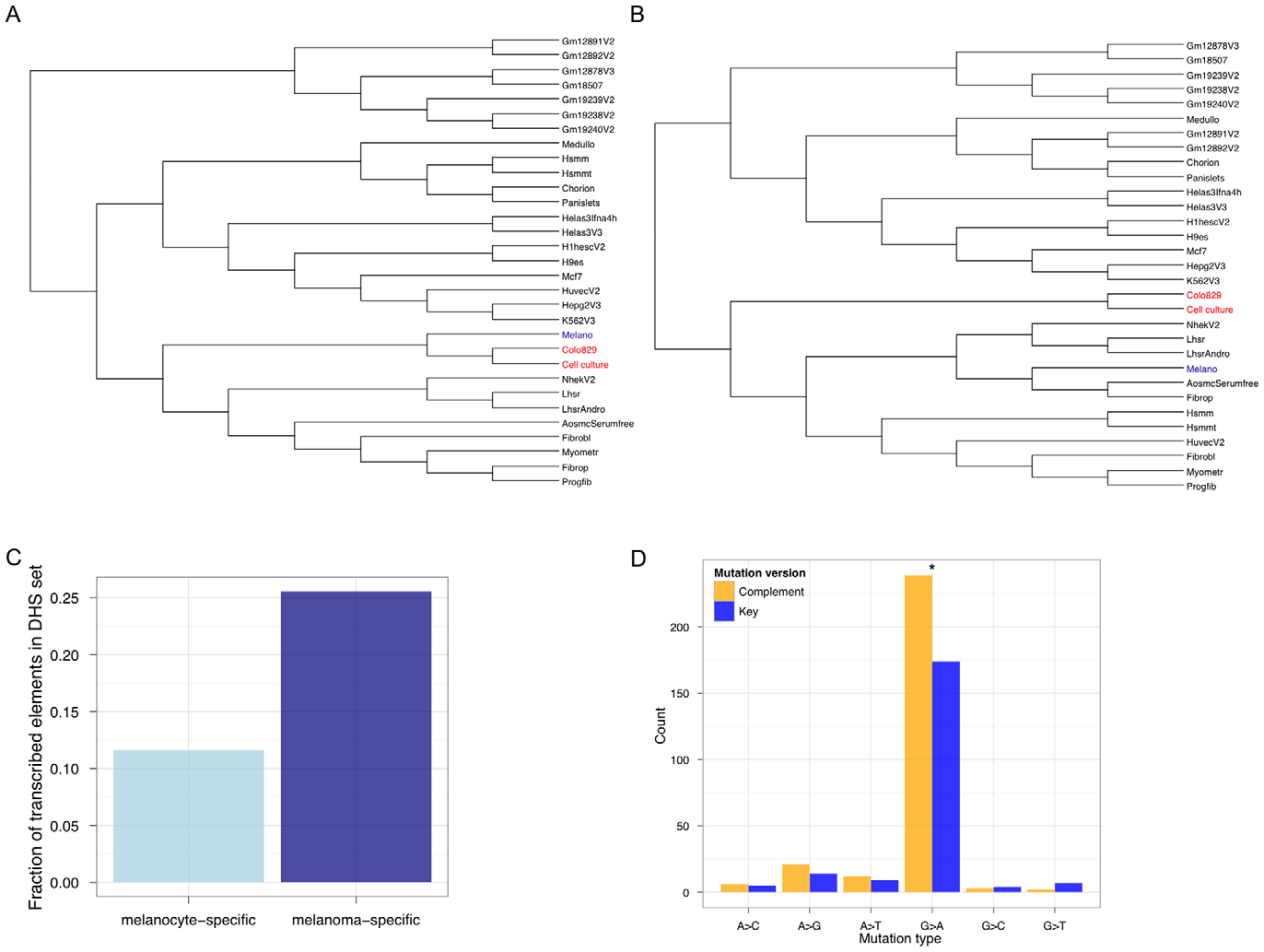
The regulatory signature of metastatic melanoma. Genome-wide DNase-Seq identifies (DHS) regulatory elements in the cell culture sample from our study and the colo-829 cell line. (A) Hierarchical clustering of all DHSs shows that the regulatory architecture of metastatic melanoma cells (red) adopts that of a more derived melanocyte (blue). (B) Focusing on exon-overlapping DHSs to identify the open chromatin landscape in gene regions shows that the metastatic melanoma cells are de-differentiated relative to melanocytes. Of the DHSs that occur in exonic regions and are specific to the metastatic melanoma samples (and not present in any others), the important melanoma genes MITF, NEDD9, and DCC are identified. (C) Melanoma transcription at melanoma-specific (dark blue) TSS-distal DHSs is significantly more frequent (P<2.2e−16; Fisher's Exact Test) than at melanocyte-specific (light blue) TSS-distal DHSs. (D) Mutational bias in melanoma DHSs is asymmetric with respect to orientation relative to the transcribed strand. The 12 possible mutations are collapsed into 6 such that the key mutation (A>C, for example; blue) and its complement (T>G; yellow) version are represented with different colors. An asterisk (*) represents P<0.05 for a Binomial test, using a 50% expectation, on the counts for a pair of key and complement mutations.

To experimentally test the hypothesis of regulatory de-differentiation, we performed genome-wide DNase-Seq to identify DHSs in colo-829 and the cell culture sample sequenced in this study. Generating trees using these two samples and DHSs from the other cell types shows that the two melanoma samples are closely related to each other and melanocytes (Figure 4A). However, focusing on gene regulatory status by only considering DHSs that overlap exonic regions shows a different tree topology (Figure 4B). Here, the melanomas are de-differentiated relative to the melanocyte sample.

It is known that highly transcribed genes accumulate fewer somatic mutations relative to more lowly transcribed genes [1]. Thus, the extent of TCR depends on the level of transcription. Recent studies show that non-coding functional elements are transcribed [33]–[36]. So, one would expect that somatic mutation accumulation in these regions could be modulated by whether or not, and to what extent, they are transcribed. To determine if TCR might operate at melanoma-specific TSS-distal non-genic regulatory regions, we calculated how many of these sites are transcribed (Figure 4C). We found that melanoma-specific regulatory regions are significantly more likely to be transcribed (P<2.2 e−16; Fisher's Exact Test) relative to melanocyte-specific regulatory regions. To further investigate this, we searched for a hallmark signature of TCR—repair events biased to the transcribed strand. Focusing on SSNVs overlapping the melanoma regulatory elements we identified that occur within introns (so that we can orient mutations relative to the transcribed strand), we observe a significant (P = 0.001605; exact binomial test) strand bias (Figure 4D). These are the first results to our knowledge that demonstrate the regulatory architecture at non-coding regions in cancer genomes is de-differentiated and likely shaped by TCR.

Here we have used whole-genome sequencing to identify the somatic mutations in a metastatic melanoma tissue sample and a low-passage cell culture derived from the same patient. We speculate that the mutational signatures in the metastatic cell indicate that regulatory architectures of the precursor cell it was derived from and other basal cellular programs were utilized during the path to metastasis—consistent with a tumorigenesis model of embryonic program redeployment.

## Materials and Methods

### Tumor tissues

A pathology-confirmed metastatic melanoma tumor resection, paired with a pheresis-collected peripheral blood mononuclear cells, was collected from a 33 year old melanoma patient enrolled in IRB-approved clinical trials at the Surgery Branch of the National Cancer Institute. A portion of the fresh tumor was frozen and embedded in Optimal Cutting Temperature (OCT) embedding medium. A melanoma cell line was derived from mechanically dispersed tumor cells, which were then cultured in RPMI 1640+10% FBS at 37°C in 5% CO_2_ for 9 passages. Genomic DNA was isolated using DNeasy Blood & Tissue kit (Qiagen, Valencia, CA). Several quality controls were performed one of which was the use of cytopathology, to determine the percentage of melanoma antigen expressing cells. The tissue culture line used in this study was evaluated by immunohistochemistry to have at least 75% of cells express melanoma-specific antigens. This threshold was set as it has been reported to give sufficient purity to identify regions of homozygous deletion, hemizygous deletion, copyneutral LOH, duplication and amplification [37]–[39]. Genotyping of the samples was performed to verify that they are derived from the same individual.

### Melanoma tissue processing for Laser Capture Microdissection (LCM)

H&E stained sections of fresh frozen melanoma tissues are prepared for initial histologic assessment. Sections are examined by a pathologist for the presence of tumor, estimation of tumor content, presence of inflammation and necrosis. Tissues with less than 70% tumor and/or significant areas of inflammation and necrosis are subjected to LCM.

### Laser Capture Microdissection (LCM)

Laser capture microdissection (LCM) was performed in the Pathology Core Facility of MSKCC, New York, NY, using the Veritas Microdissection System (Arcturus). The Veritas system combines ultraviolet laser cutting and laser capture using an infrared laser source. Fresh frozen melanoma tissues sectioned between 8 and 10 µm were transferred to PEN membrane slides (MDS Analytical Technologies) and sections were stained by using a modified protocol described previously [40], [41]. Briefly, sections were stained with hematoxylin as follows: slides were immersed in 70% ethanol for about 10 min followed by sequential dips in nuclease free water, Mayer's hematoxylin solution for 30 sec, nuclease free water, 75% ethanol, 95% ethanol and finally dehydrated in absolute ethanol by 3 changes of 3 min each.

Multiple serial sections (10–20) of the tissue are used to maximize cell yields. 5,000 to 10,000 cells were harvested in each LCM cap and material from 5–10 caps was pooled together to maximize yields.

### DNA extraction

DNA was extracted using DNeasy Blood and Tissue kit (Qiagen) following manufacturer's instructions. DNA was eluted in 35 ul of elution buffer. DNA measurements were made using ND-1000 UV-Vis spectrophotometer from NanoDrop technologies.

### Genome build statistics

We generated 5,409,104,173 100 base paired-end reads that pass the Illumina chastity filter and contain 32 or more Q20 Sanger-scaled quality bases for this study, which were partitioned among the genomes as follows: 1,042,502,044 for the cell culture, 1,588,246,159 for the tissue, and 2,778,355,970 for the normal. Reads were aligned to the unmasked hg18 version of the human genome using BWA [42] with default parameters. After removing molecular duplicate read pairs (read pairs that map to the same position on the reference sequence are likely an artifact of sample preparation) using samtools [43] and considering only reads with a mapping quality of Q30 or greater and bases with quality of Q20 or greater, we observe an average base coverage of 21.4×, 29.6×, and 47.7× for the cell culture, tissue, and normal genomes, respectively (Figure S1). Within coding regions, we were able to make confident variant calls (see details below) at 64.3%, 85.2%, and 88.9% of the positions in the cell culture, tissue, and normal genomes, respectively. Comparing territory that is callable in the cell culture and tissue results in 81.1% genome coverage.

### Single nucleotide variants

For variant calling, only reads with mapping quality of Q30 or greater and bases with quality of Q20 or greater were considered. We used two related algorithms to make single-position genotype calls in the normal and melanoma genomes. For all genomes, we use a Bayesian genotype caller named Most Probable Genotype (MPG) that has been described previously [44]. This genotype caller produces accurate calls in regions that satisfy whole-genome coverage and quality parameters as determined by a separate study [7]. Namely, the MPG score must be equal or greater than 10 and the MPG score to base Q20 quality-coverage ratio must be equal to or greater than 0.5. To independently verify MPG calls, we compared genotypes to those called by the Infinium 1M quad SNP-chip platform. The genotype concordance rate with the SNP-chip for the normal genome is 99.937% at 99.3% of the positions, excluding regions with hidden SNPs [45] and abnormal copy number. A similar comparison performed on the cell culture genome results in 99.939% concordance at 91.2% of the positions. To better identify variant positions in the cell culture and tissue genomes, we first developed a new algorithm similar to MPG, called Most Probable Variant (MPV). An important distinction between MPG and MPV is that the MPV score reflects the degree of confidence that a sample has a genotype different from the reference genome, whereas the MPG score reflects the degree of confidence in the genotype call itself. MPV is a new option (–score_variant) in the MPG program and the executable source code is freely available for download from the following URL: http://research.nhgri.nih.gov/software/bam2mpg/. We optimized calling parameters for MPV by downloading and analyzing genome-wide tumor and normal data that was previously published for the colo-829 melanoma and colo-829BL normal cell lines [1]. Using MPV with optimized parameters (MPV score must be greater than or equal to 10 with no coverage ratio criteria similar to the MPG parameters) on the cell culture genome allows us to identify more variant positions without dramatically sacrificing accuracy (Table S2). Comparing MPV calls for the cell culture genome to the SNP-chip results in 99.79% concordance at 96.28% of the variant positions.

To identify novel somatic single nucleotide variant (SSNV) positions we compared the MPV-called genotype in either melanoma genome to the MPG-called genotype in the normal genome and then subtracted out any variants that are present in dbSNP129 or within ten bases of an indel identified by the MPV algorithm. Loss of heterozygosity (LOH) variants were ignored since there is no novel somatically-acquired allele.

Running our analysis pipeline on our own samples resulted in 122,837 SSNVs in the cell culture genome and 105,460 in the tissue genome. It is important to note that these two numbers are not comparable because they are not normalized across the common callable territory in the cell culture and tissue genomes. Once we account for this, the somatic variant counts drop to 97,532 for the cell culture and 98,548 for the tissue.

We validated novel SSNVs by PCR amplifying the regions in the cell culture and normal genomes and then Sanger sequencing the products. Of 192 randomly chosen positions (96 in coding regions, and 96 in non-coding regions), we were able to successfully PCR amplify and Sanger sequence 181 in both genomes. Of these, we observed evidence for somatic variants concordant with the whole-genome data at 100% of the positions.

For further validations we randomly selected 96 cell culture-specific and 96 tissue-specific SSNVs for PCR amplification and Sanger sequencing. Of the 78 successful PCR and Sanger sequencing reactions for the cell culture set, 75 (96%) had genotype calls concordant with the whole-genome sequencing call. For the tissue set, 43/73 (59%) were concordant. This result allowed us to focus on the 30 positions where the tissue-specific whole-genome calls were not concordant with the PCR and Sanger calls. We found that by implementing three simple filters, we eliminated 29 of 30 discordant positions and 0 of 43 concordant positions, so that the concordance rate is 43/44 (97.7%). The filters we implemented are:

1. Normal lookup filter to check for somatic variant alleles in the normal genome, as previously described [5].
2. Indel filter to remove somatic variant calls within 10 bases of an indel call made using reads with a mapping quality of 1 or greater and a MPV score of 10 or greater in the tumor genome.
3. Strand bias filter to remove calls where the somatic allele is present exclusively in reads mapping to one strand and not the other.

These filters removed 2 of 3 discordant cell culture-specific calls and 0 of 75 concordant calls, so that the concordance rate is 75/76 (98.7%).

We additionally looked at the mutation spectrum for all the common, tissue-specific, and cell culture-specific SSNVs (Figure S5). All three mutation spectrums are enriched for the known G>A/C>T UV signature. Together, these results suggest that our method is highly specific.

### Cellular heterogeneity

To estimate the extent of normal cell contamination in the cell culture and tissue samples, we calculated the fraction of reads with mapping quality of at least 30 supporting the acquired somatic allele at heterozygous positions and compared this to what would be expected in a completely homogenous cellular population with no normal cells. Our analyses show that the cell culture has no normal contamination, while the tissue sample contains about 42% normal cells (Figure S17). Other groups have used similar methods to estimate tumor sample purity [46].

### Copy number variants

We first estimate copy number in non-overlapping 5 Kb tiles in the normal genome using the copySeq algorithm [8]. We only consider tiles with 80% uniquely mappable k-mers (which is 94% of the tiles) to ensure accurate copy number estimation. To detect amplifications and deletions in the cancer genomes we use the CNV-seq algorithm [9], which compares the cancer to normal genome, with the following parameters:


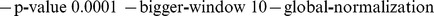


Somatic copy number alterations (SCNAs) are then defined over the 5 kb tiles called in the normal genome using CNV-seq results and copy number is adjusted based on the level of normal genome contamination, as described above. Adjacent amplified or deleted 5 kb windows are merged and only regions where two or more windows are affected are retained. To conservatively identify tissue or cell culture-specific CNVs, we filtered the CNV-seq calls in one sample by looking at the corresponding log2 ratio in the other sample. Any CNV-called regions in one sample with a CNV log2 ratio< = −0.1 or > = 0.1 in the other sample were considered CNVs even if they were not called by the CNV-seq algorithm. Thus, these regions are not considered sample-specific, which result in a conservative set of CNV calls.

### Somatic insertions and deletions

We made somatic insertion and deletion calls by extending our MPV and MPG scoring methodology (see Single nucleotide variants section above) to indel calls. We first select all possible non-reference indel calls, irrespective of score threshold, across all three genomes using MPG on the normal genome and MPV on the tumor genomes. After merging all possible calls, we then look at each genome independently and determine how well the reads support the indel call using MPG on the normal genome and MPV on the tumor genome, both with thresholds of 10. To find somatic indels we keep non-reference tumor calls that do not match the normal call at that same position. Because CNVs can bias indel calls, we subsequently filter by retaining regions where the CNV log2 ratio >−0.1 and <0.1 such that a conservative set of indels outside CNV regions are compared. The final indel results are summarized in Figure S6.

### Array comparative genome hybridization (aCGH)

We used an Agilent 180K aCGH array to look for CNVs in the tissue sample. For gain/loss calls, we used the default Nexus 6.0 settings for Agilent 180K catalog arrays for mosaic tissue samples, and adjusted the minimum probe bin size to 10 instead of the default 3 for segmentation

### Chromosomal rearrangements

We used the BreakDancer algorithm [10] to detect chromosomal rearrangements. In order to detect somatic events we require a score of 90 or greater in the cancer genome, which is consistent with parameters reported in a previous study [6] and no evidence in the normal genome. We further filter the results in two ways. First, by removing any somatic events that occur in any of ten normal genomes from an ongoing internal study (data not shown). Second, by requiring that the 2 kb region immediately surrounding each putative breakpoint is greater than 99% mappable according to the CRG 100mer alignability track available at the UCSC genome browser.

### Mutation accumulation enrichment

We used the Genome Structure Correction (GSC) method [47] to calculate enrichment statistics for SSNVs relative to other genomic features. All results are based on 10000 samplings and reported as the log2 fraction of observed base overlaps divided by the mean of the null overlaps. Error bars represent +/− one standard deviation from the mean of the null distribution. We calculate enrichment or depletion only in situations where ten or more SSNVs overlap a particular set of genomic features. If there are fewer overlaps, we consider the calculation unreliable and therefore ignore those comparisons. Common SNP control data sets were constructed using 1000 Genomes calls [48] at positions that have a minimum of 5% minor allele frequency (MAF) and are concordantly called across four different centers. Similar results are observed when using 20% MAF SNPs (data not shown).

### Chromatin segmentations

Chromatin segmentation data for nine different cell types was obtained from Ernst et al. [24]. We ignored states 14 and 15, which correspond to repetitive regions of the genome. Variant calls are generally filtered out of these areas by the 1000 Genomes Consortium because they result in high false positives rates. These two states combined occupy 0.27% of the genome on average over the nine different cell types, so ignoring them will have little effect on our analyses.

### Hierarchical gene-landmark partitioning

We divided genomic features into hierarchical and mutually exclusive categories based on the following hierarchical sequence of genic landmarks: coding regions, 5′ UTR's, 3′ UTR's, introns, intergenic transcription start site (TSS)-proximal (within 5,000 bp of a TSS), and intergenic TSS-distal (greater than 5,000 bp from a TSS). All genic landmarks are based on the GENCODE annotation [49] in hg18 and can be downloaded from the UCSC Genome Browser [genome.ucsc.edu].

### Non-genic tiles

We masked out all regions of the genome overlapping with, or within 10,000 bp, of any part of a gene or TSS. For the remaining parts of the genome, we created 50,000 bp non-overlapping tiles and calculated the number of bases that overlap evolutionarily constrained regions. Constrained regions are based on the GERP method [20] and the Enredo, Pecan, Ortheus (EPO) alignments [50], [51] and are available at the Ensembl browser [www.ensembl.org]. We discarded tiles with no constrained region overlap and sorted the remaining tiles by the fraction of constrained base overlaps. Using this sorted list, we created ten equal-sized bins and calculated mutation accumulation enrichment (see above) for the tiles within each bin.

### DNase I Hypersensitive Site (DHS) data sets and analysis

We used post-embargo ENCODE Consortium DHS data sets for the following 29 cell lines: AosmcSerumfree, Chorion, Fibrobl, Fibrop, Gm12878, Gm12891, Gm12892, Gm18507, Gm19238, Gm19239, Gm19240, H1hesc, H9es, Helas3Ifna4h, Helas3, Hepg2, Hsmm, Hsmmt, Huvec, K562, Lhsr, LhsrAndro, Mcf7, Medullo, Melano, Myometr, Nhek, Panislets, Progfib. Information about the cell lines and DHS experiments can be found at the UCSC ENCODE Open Chromatin, Duke/UNC/UT Track Settings Page: http://genome.ucsc.edu/cgi-bin/hgTrackUi?g=wgEncodeChromatinMap.

Enrichment statistic measurements and gene-landmark partitioning for DHS regions were performed as described above. Single-linkage clustering was performed on all DHSs across the 29 cell lines to determine regions that are active in single, multiple, and all cell types. The DHS signature tree was constructed by first creating a binary vector for each cell type that classifies a region as either on (1) or off (0). Then, Euclidean distance was used as a metric to hierarchically cluster the binary vectors. The resulting trees were manipulated with the Dendroscope program [52] to re-root using the GM cell types as an out group. Figure 4 reports the result of this analysis on all DHSs, but we observe the same tree topology when only non-genic TSS-distal DHSs are considered (data not shown).

### Experimental DHS identification in melanoma samples

DNase-seq libraries we generated as previously described [29], [30] and sequenced via Illumina's GAII sequencer. After alignment to the human reference sequence, we used F-seq [53] to identify DHS peaks. These peaks were compared to DHS regions identified in the same manner from other cell types.

### Transcription at DHSs

We used transcribed regions from ten melanoma samples as defined by Berger et al. [25]. A DHS element is considered transcribed if any high mapping quality (mapQ> = 30) RNA-seq read from any of the ten melanoma samples overlaps the DHS.

## Supporting Information

Figure S1 Reference genome coverage for all three samples using reads with a mapping quality of Q30 or greater and bases with a base quality of Q20 or greater.
(TIF)

Figure S2 Somatic alterations in the tissue (A) and cell culture (B) genomes. Whole-genome SSNV, SCNA, and translocation results are presented for each sample. Blue bars represent the number of SSNVs per 10 Mb. Interior to the blue bars, blue lines on a gray background represent SCNAs from copy one to five. Inside the circle, red and gray lines represent interchromosomal and intrachromosomal translocations, respectively.
(TIF)

Figure S3 Somatic copy number alterations (SCNAs) called using the whole-genome data have a high degree of concordance with SCNAs called using aCGH data.
(TIF)

Figure S4 Copy number variation (CNV) differences in the cell culture and tissue genomes relative to the normal genome. In some instances, tissue CNV regions appear to nucleate larger CNV events in the cell culture.
(TIF)

Figure S5 The mutational spectrum for all common, cell culture-specific, and tissue-specific SSNVs.
(TIF)

Figure S6 Somatic indel calls in the tissue and cell culture samples. (A) After CNV filtering there are 269 shared somatic indel events, while the tissue (blue) has 127 unique events and the cell culture (yellow) has 160 unique events. (B) Somatic indel size counts show that smaller indels (around size +1 or −1) are more common than larger events.
(TIF)

Figure S7 A comparison of SSNVs called on the colo-829 using the method presented here and the method originally described by Pleasance et al. [1].
(TIF)

Figure S8 The mutational spectrum for SSNVs called on the colo-829 genome.
(TIF)

Figure S9 Shared genic mutations among the samples. Numbers indicate the count of genes with a nonsynonymous or stop mutation. These numbers reflect variants at all callable positions per genome, not normalized across commonly callable territory. Genes with star superscripts are implicated in melanoma pathogenesis by other studies.
(TIF)

Figure S10 Variant enrichment in chromatin segmentations across nine different cell types.
(TIF)

Figure S11 Variant enrichment in chromatin segmentations across nine different cell types using samples analyzed independently.
(TIF)

Figure S12 Somatic mutation enrichment compared to fraction of evolutionarily constrained bases in chromatin segmentations across nine different cell types. R values represent Spearman's correlation.
(TIF)

Figure S13 Somatic mutation enrichment compared to fraction of coding bases in chromatin segmentations across nine different cell types. R values represent Spearman's correlation.
(TIF)

Figure S14 Regions that are heterochromatin low signal zones (state 13) accumulate somatic mutations at a rate similar to random expectation.
(TIF)

Figure S15 Mutation accumulation increases with distance along known transcripts. Each point represents a 5 Kb bin.
(TIF)

Figure S16 Genic partitioning of melanocyte DHSs such that every DHS occurs in a single category shows that most categories are depleted for mutation accumulation (TSS P = Transcription Start Site Proximal [within 5 Kb]; TSS D = Transcription Start Site Distal [greater than 5 Kb]). Common SNPs are based on 1000 Genomes calls that have at least 5% minor allele frequency (MAF). In addition to a union analysis, each sample is also analyzed independently in this plot.
(TIF)

Figure S17 Normal cell contamination levels are different in the cell culture (A) and tissue (B) samples. We measured the fraction of MapQ30 reads that support the somatic allele at heterozygous positions and compared this to a binomial distribution fitted to the observed read counts. As expected, the cell culture has no normal cell contamination, but the tissue sample does. Based on location of the observed tissue peak at 0.29 relative to the expected peak at 0.5, we estimate the tissue sample contains approximately 42% ((0.5–0.29)*2*100) normal cells.
(TIF)

Table S1 Coordinates, gene names, and amino acid changes (where applicable) for all genic mutations in the cell culture and tissue sample.
(XLS)

Table S2 We compared the genotype calls made on the whole genome data using the MPG and MPV algorithms to SNP-chip calls made on the same samples. Three sets of positions were considered: 1) all SNP-chip positions, 2) hidden SNPs (positions on the SNP-chip where a nearby SNP could affect probe hybridization) removed, and 3) hidden SNPs and CNV abnormal regions removed.
(XLS)
