# Supplementary figures and images for: Mutational Signatures of De-Differentiation in Functional Non-Coding Regions of Melanoma Genomes
Source: PLoS Genet. 2012 Aug 9;8(8):e1002871. doi: 10.1371/journal.pgen.1002871 (PMC3415438; doi:10.1371/journal.pgen.1002871)

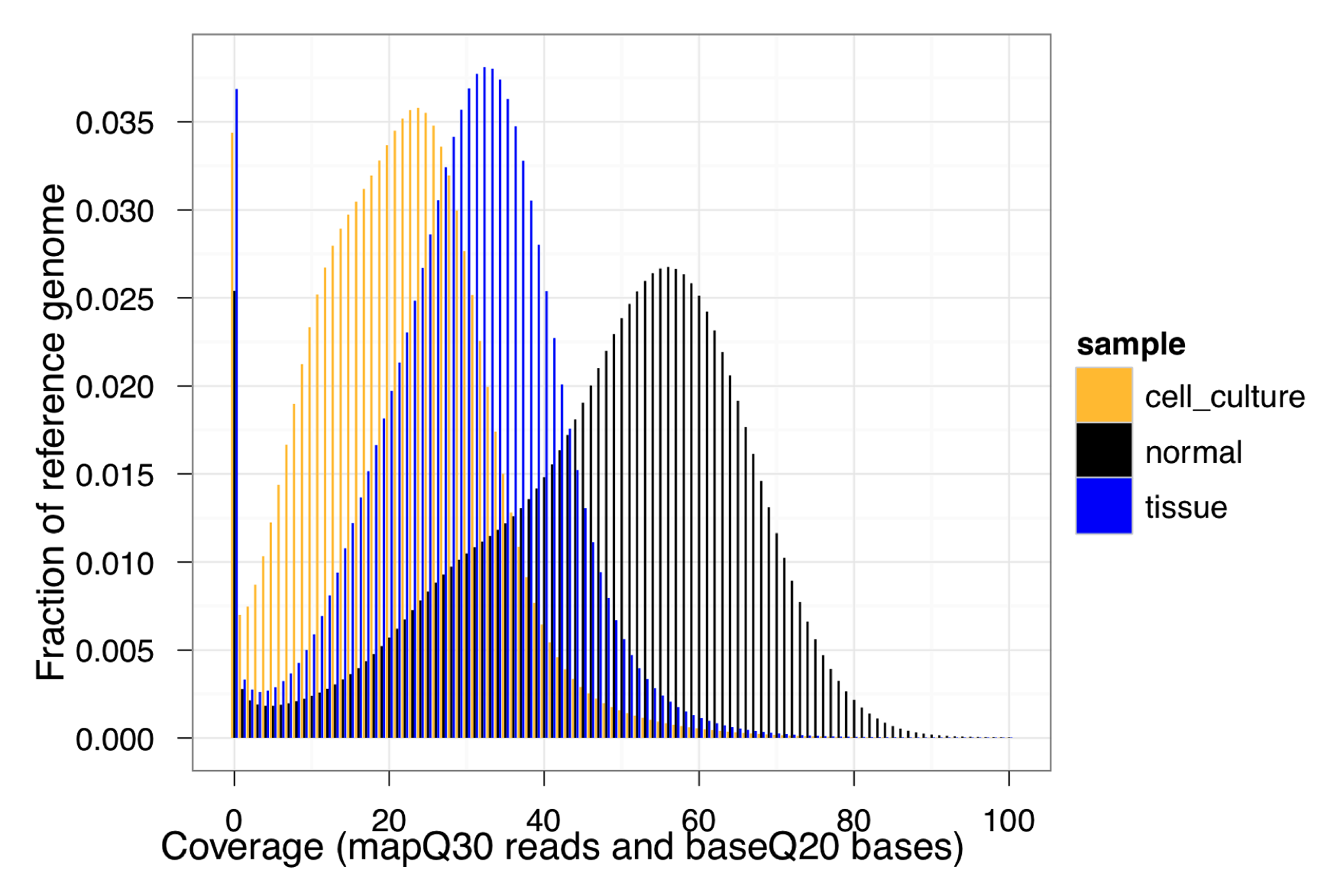

Supplement: Figure S1 — Reference genome coverage for all three samples using reads with a mapping quality of Q30 or greater and bases with a base quality of Q20 or greater. (TIF) [file pgen.1002871.s001.tif]

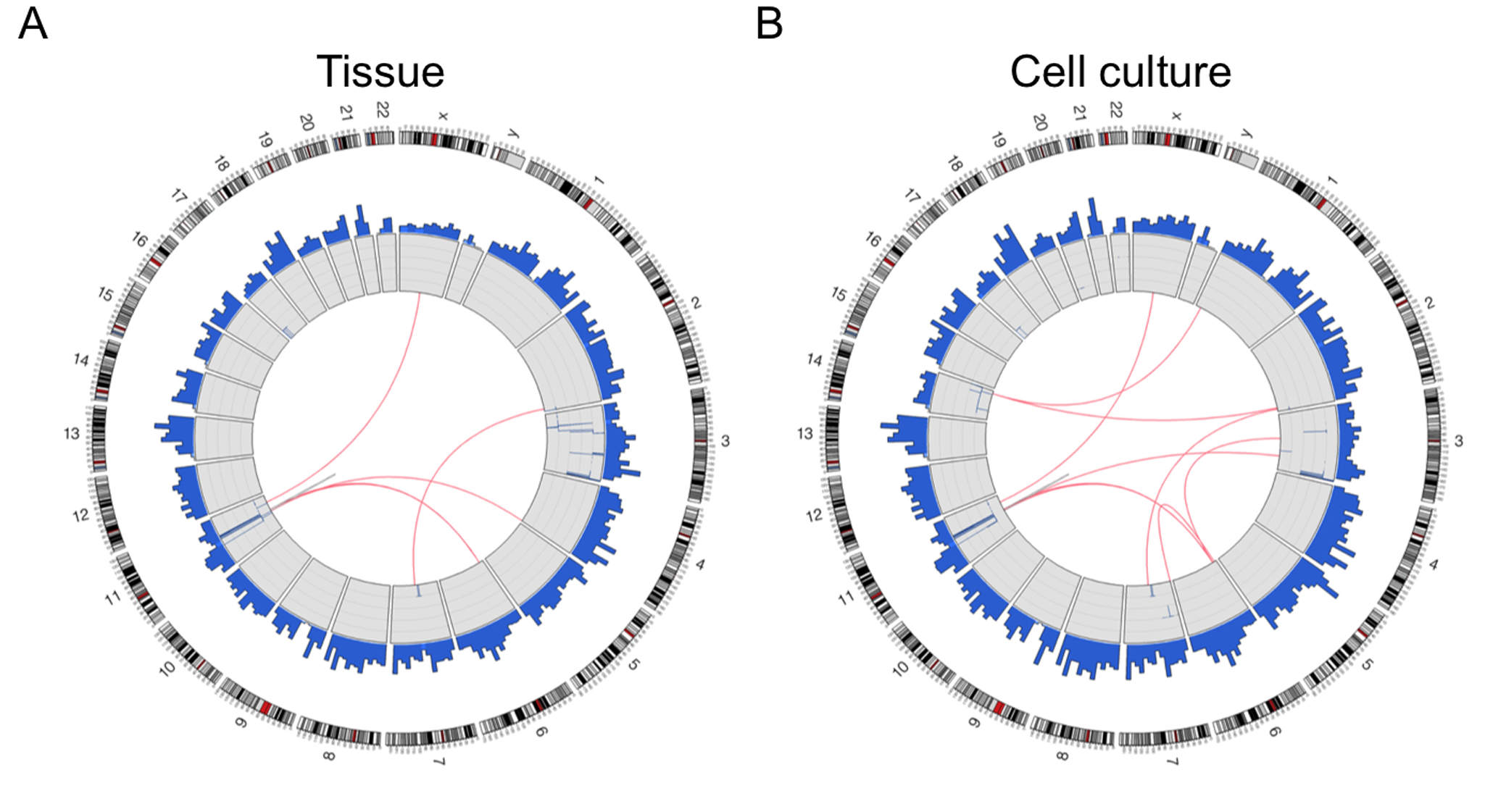

Supplement: Figure S2 — Somatic alterations in the tissue (A) and cell culture (B) genomes. Whole-genome SSNV, SCNA, and translocation results are presented for each sample. Blue bars represent the number of SSNVs per 10 Mb. Interior to the blue bars, blue lines on a gray background represent SCNAs from copy one to five. Inside the circle, red and gray lines represent interchromosomal and intrachromosomal translocations, respectively. (TIF) [file pgen.1002871.s002.tif]

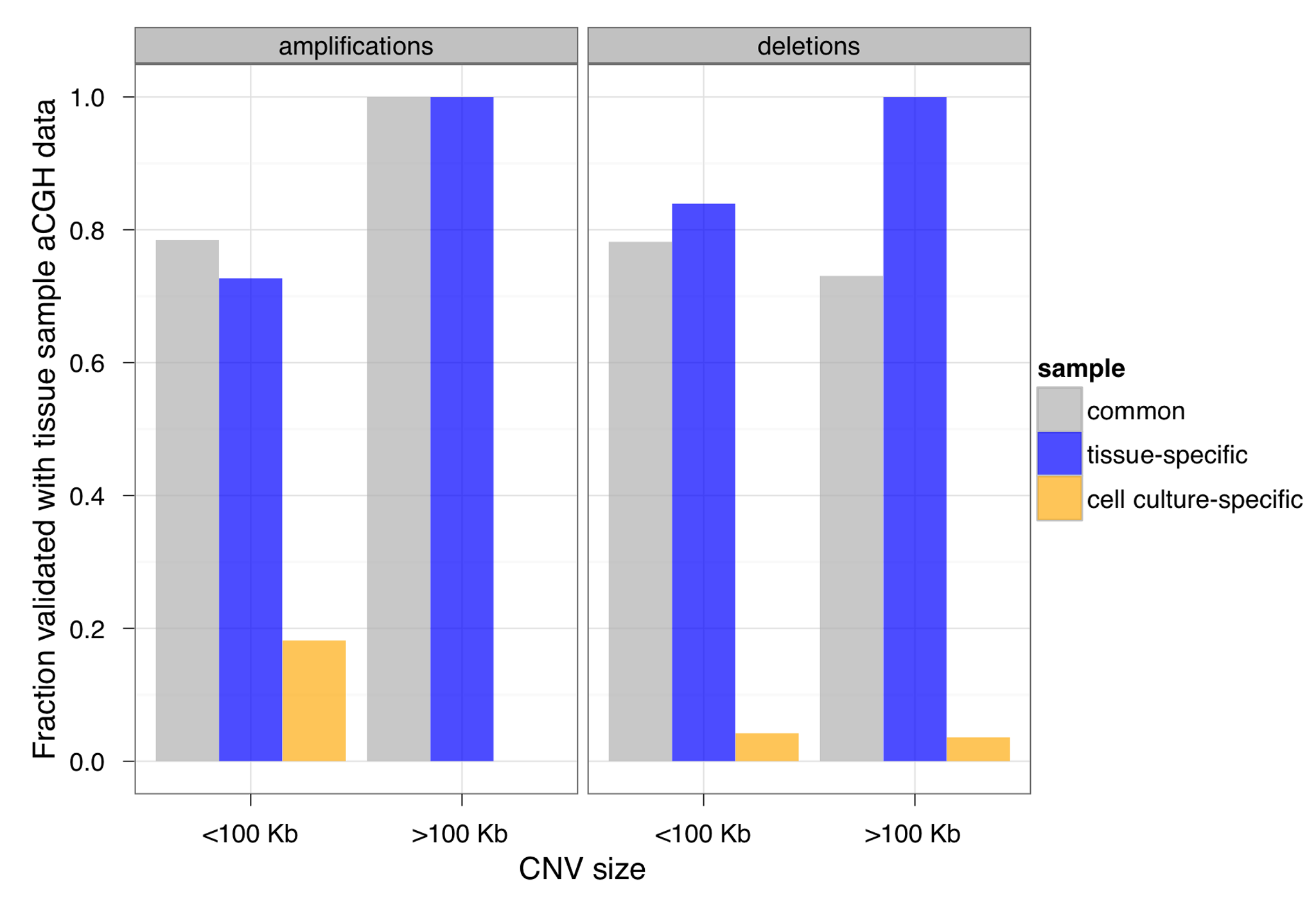

Supplement: Figure S3 — Somatic copy number alterations (SCNAs) called using the whole-genome data have a high degree of concordance with SCNAs called using aCGH data. (TIF) [file pgen.1002871.s003.tif]

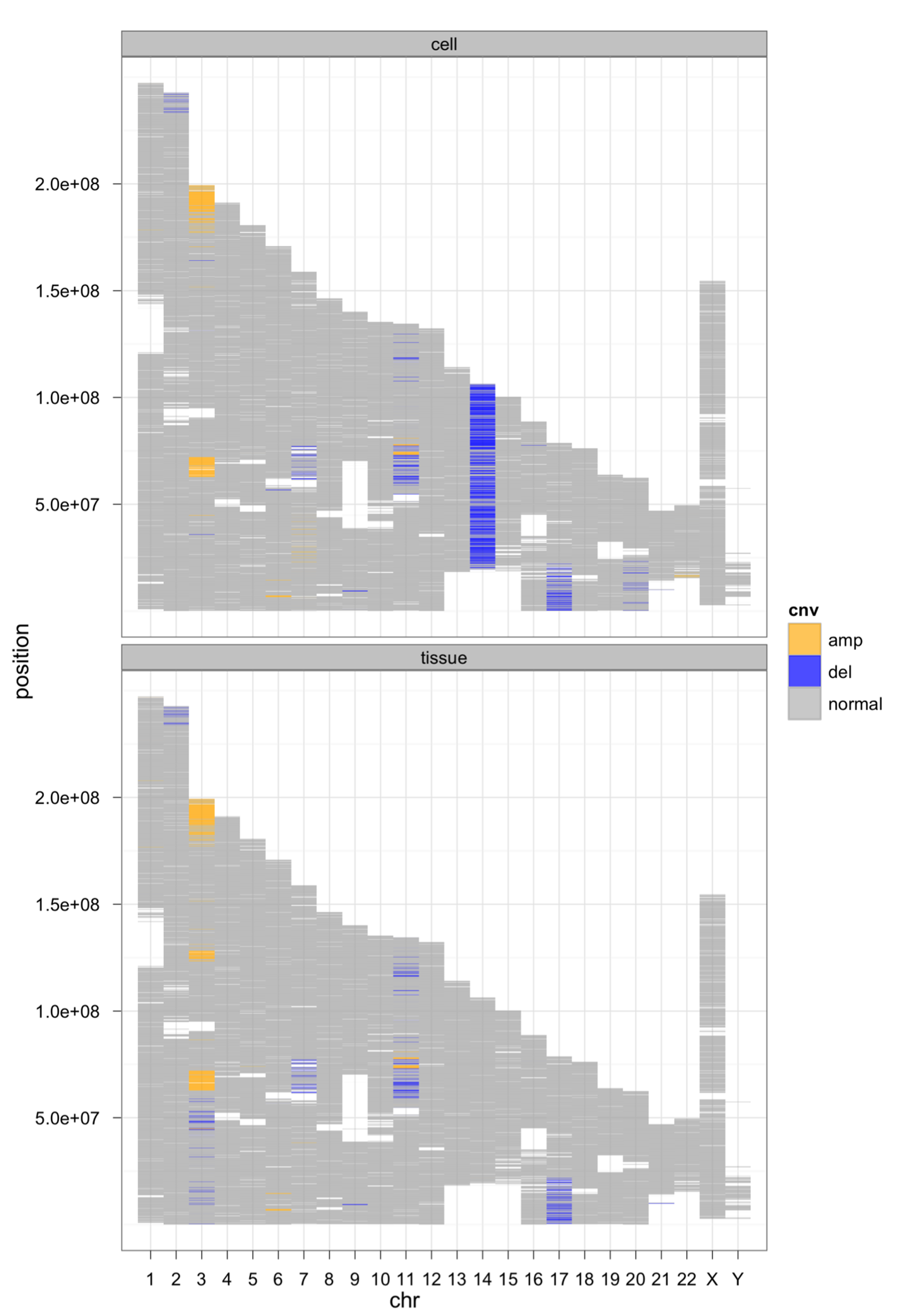

Supplement: Figure S4 — Copy number variation (CNV) differences in the cell culture and tissue genomes relative to the normal genome. In some instances, tissue CNV regions appear to nucleate larger CNV events in the cell culture. (TIF) [file pgen.1002871.s004.tif]

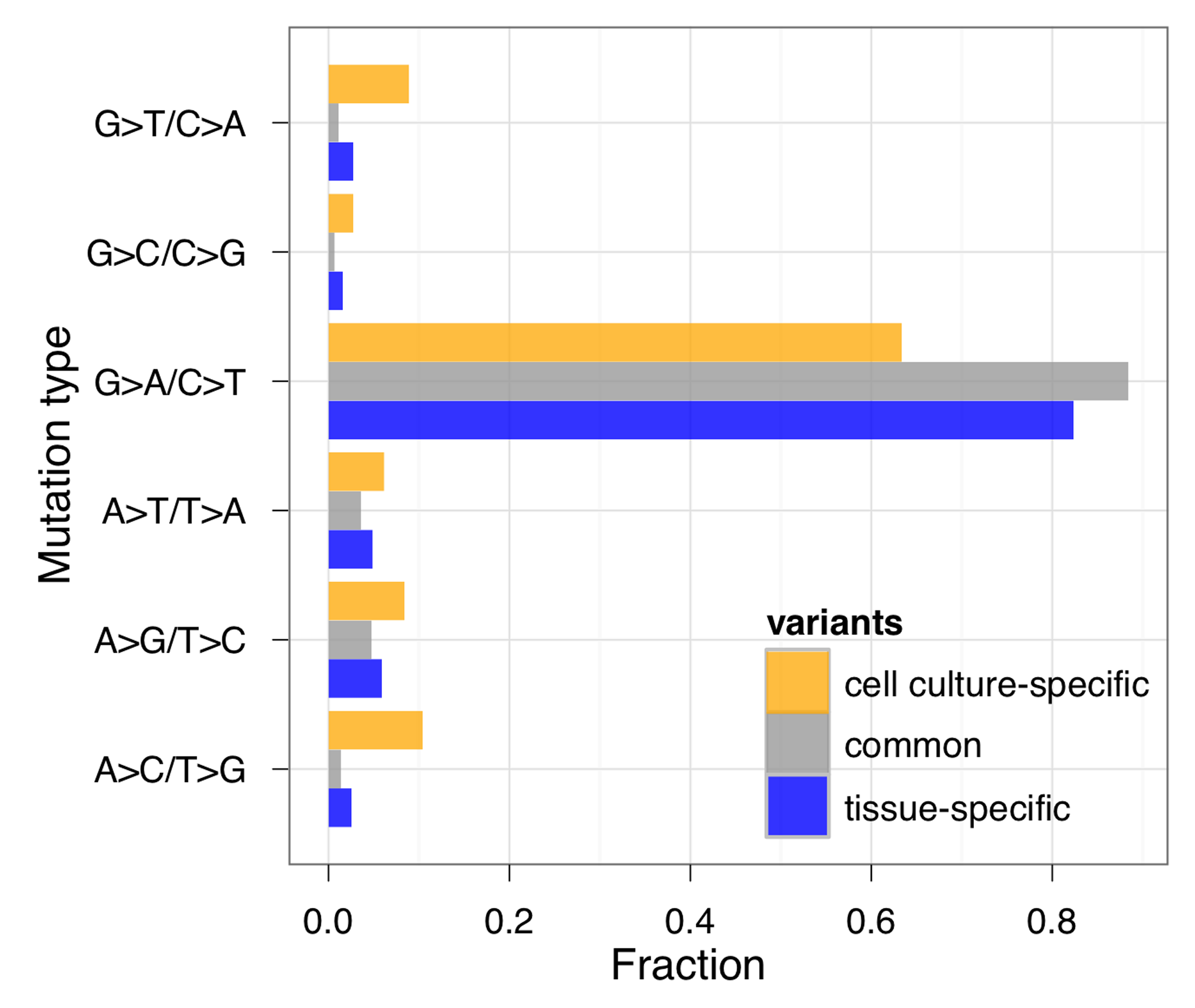

Supplement: Figure S5 — The mutational spectrum for all common, cell culture-specific, and tissue-specific SSNVs. (TIF) [file pgen.1002871.s005.tif]

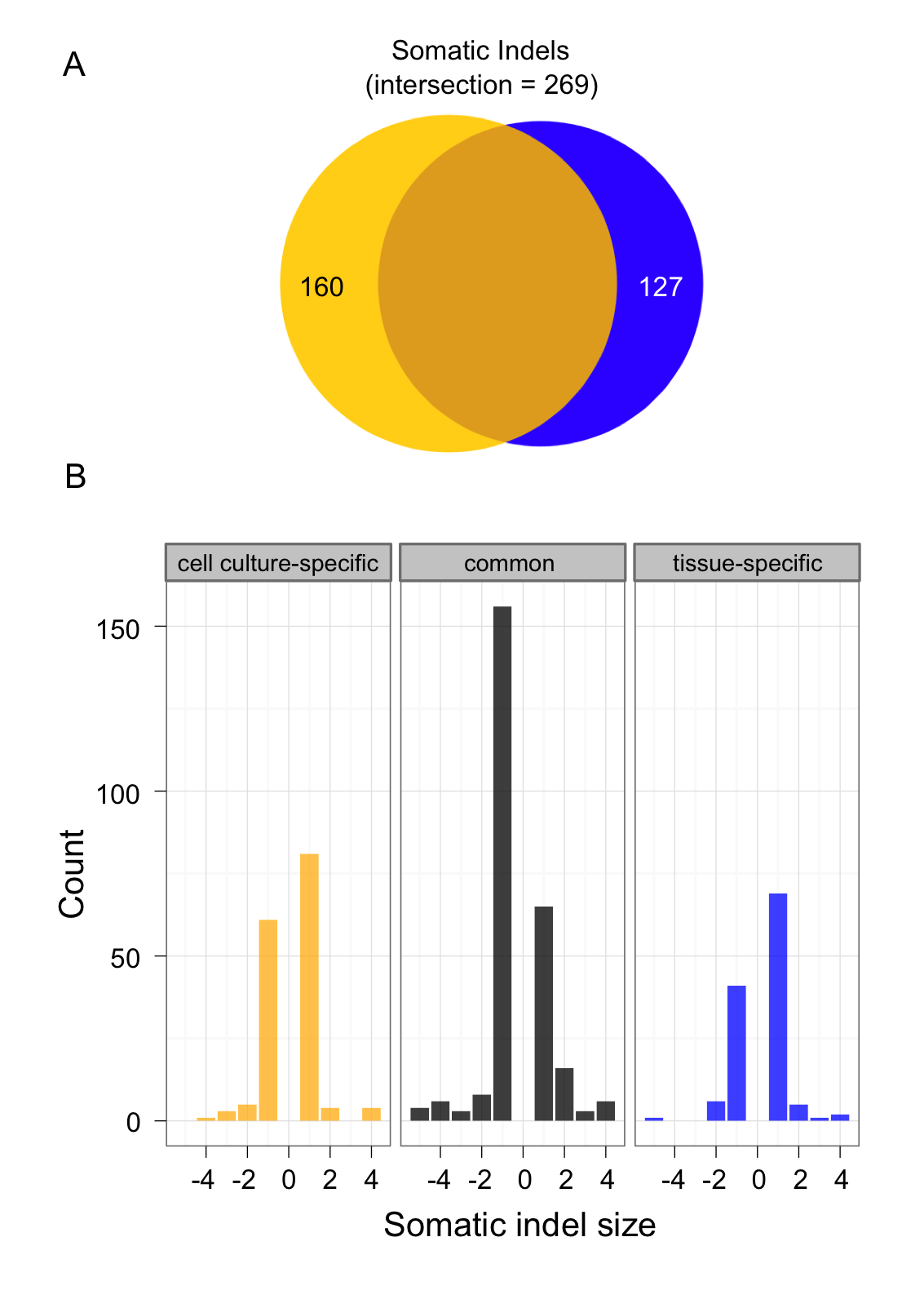

Supplement: Figure S6 — Somatic indel calls in the tissue and cell culture samples. (A) After CNV filtering there are 269 shared somatic indel events, while the tissue (blue) has 127 unique events and the cell culture (yellow) has 160 unique events. (B) Somatic indel size counts show that smaller indels (around size +1 or −1) are more common than larger events. (TIF) [file pgen.1002871.s006.tif]

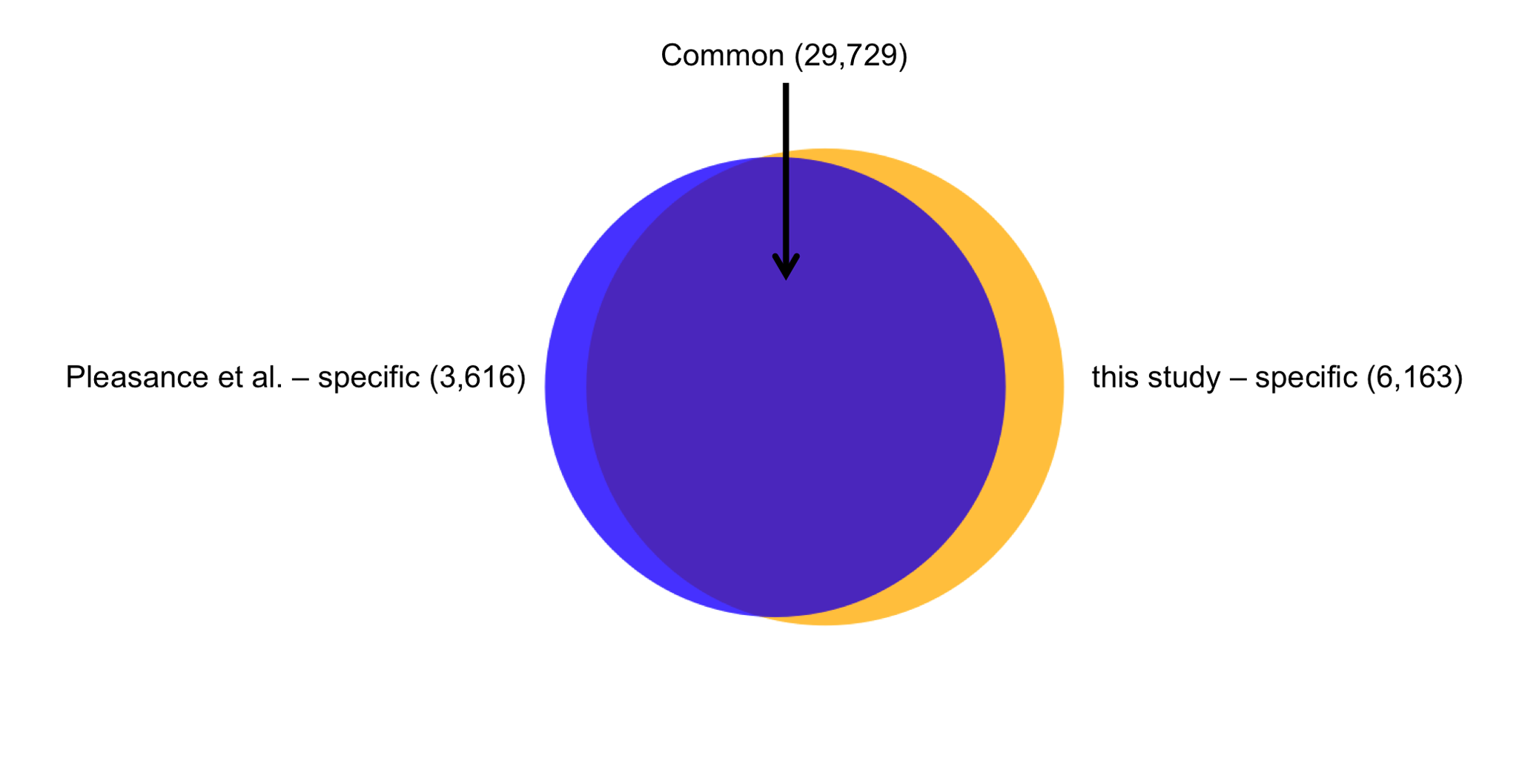

Supplement: Figure S7 — A comparison of SSNVs called on the colo-829 using the method presented here and the method originally described by Pleasance et al. [1]. (TIF) [file pgen.1002871.s007.tif]

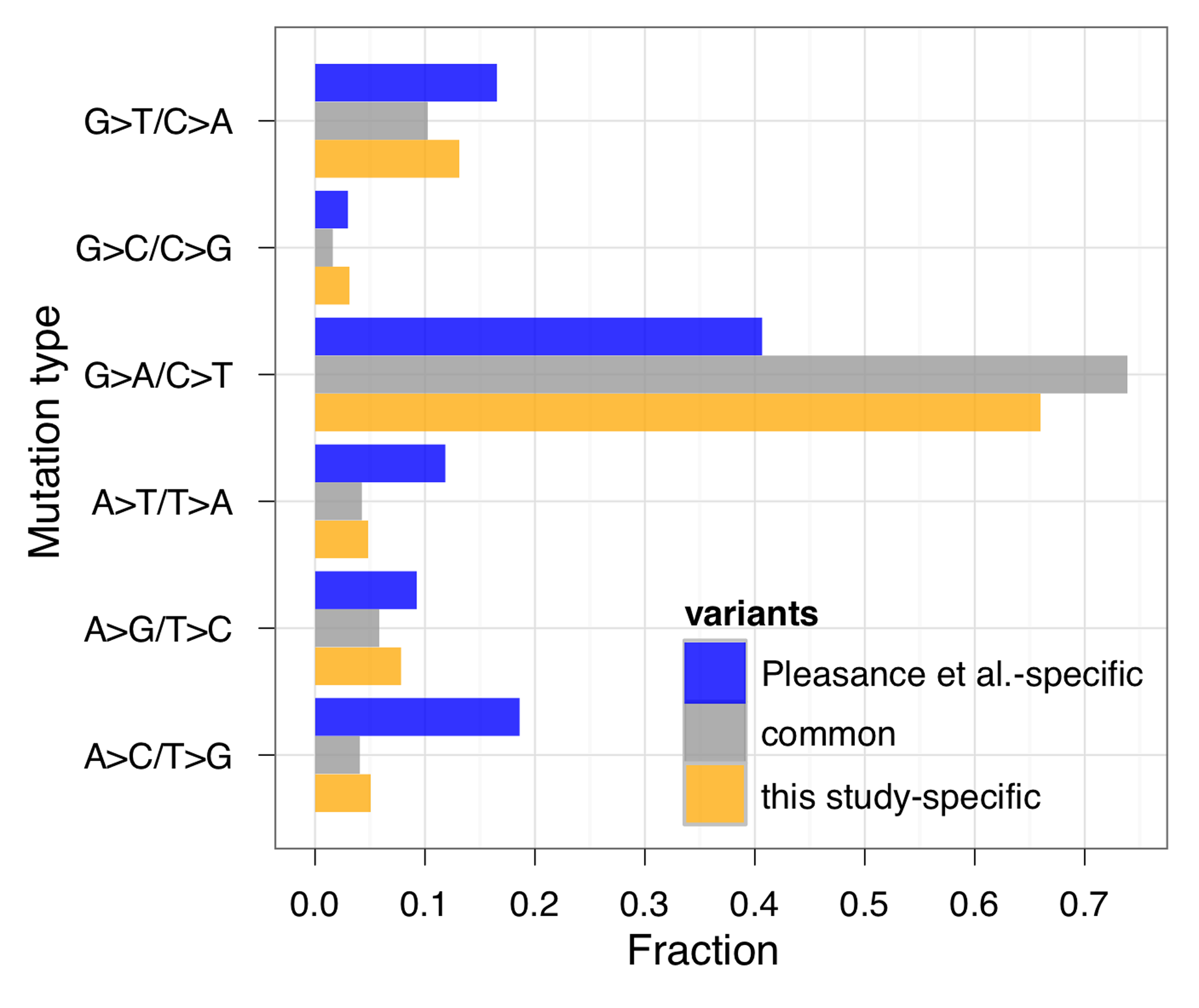

Supplement: Figure S8 — The mutational spectrum for SSNVs called on the colo-829 genome. (TIF) [file pgen.1002871.s008.tif]

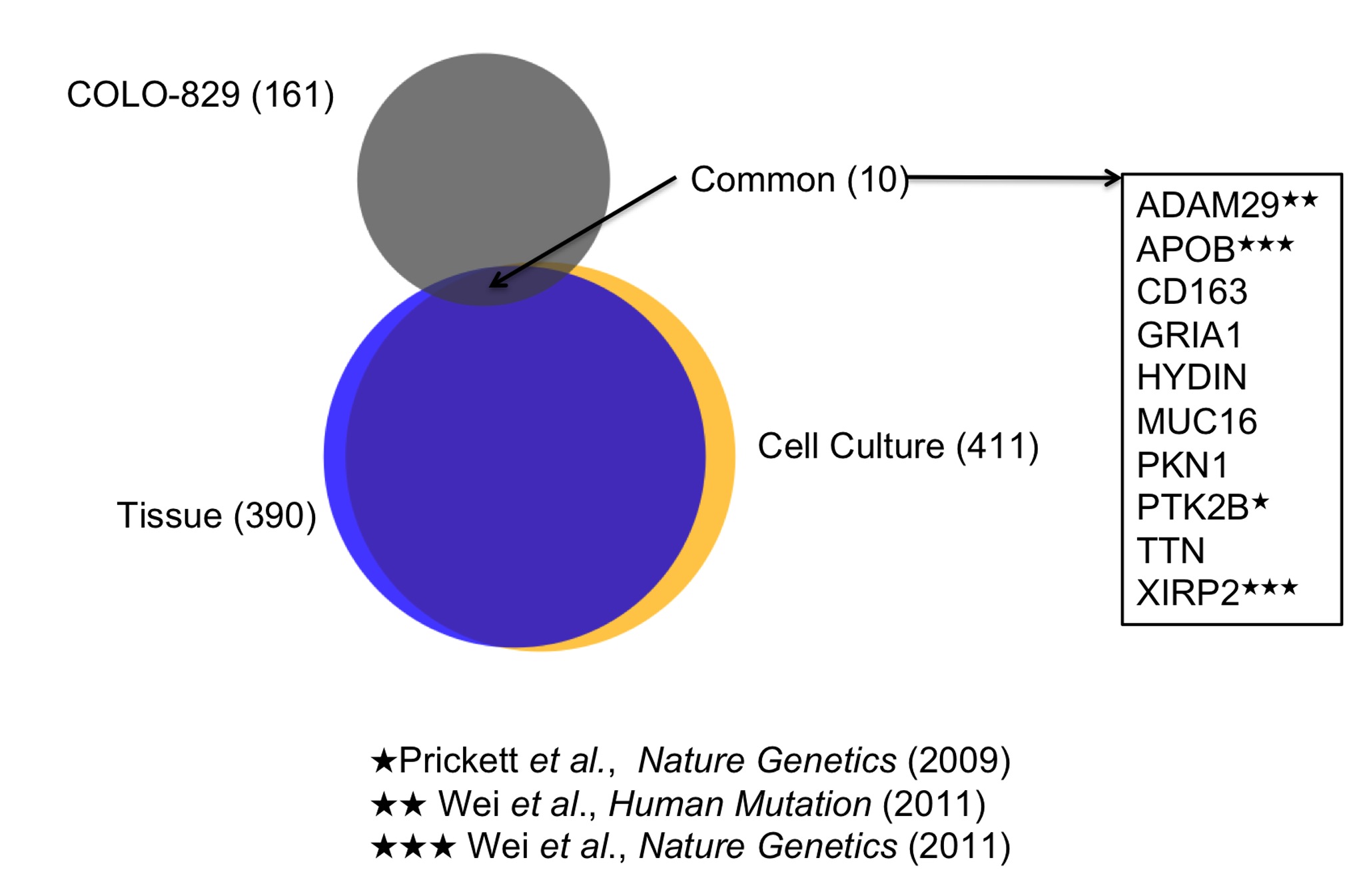

Supplement: Figure S9 — Shared genic mutations among the samples. Numbers indicate the count of genes with a nonsynonymous or stop mutation. These numbers reflect variants at all callable positions per genome, not normalized across commonly callable territory. Genes with star superscripts are implicated in melanoma pathogenesis by other studies. (TIF) [file pgen.1002871.s009.tif]

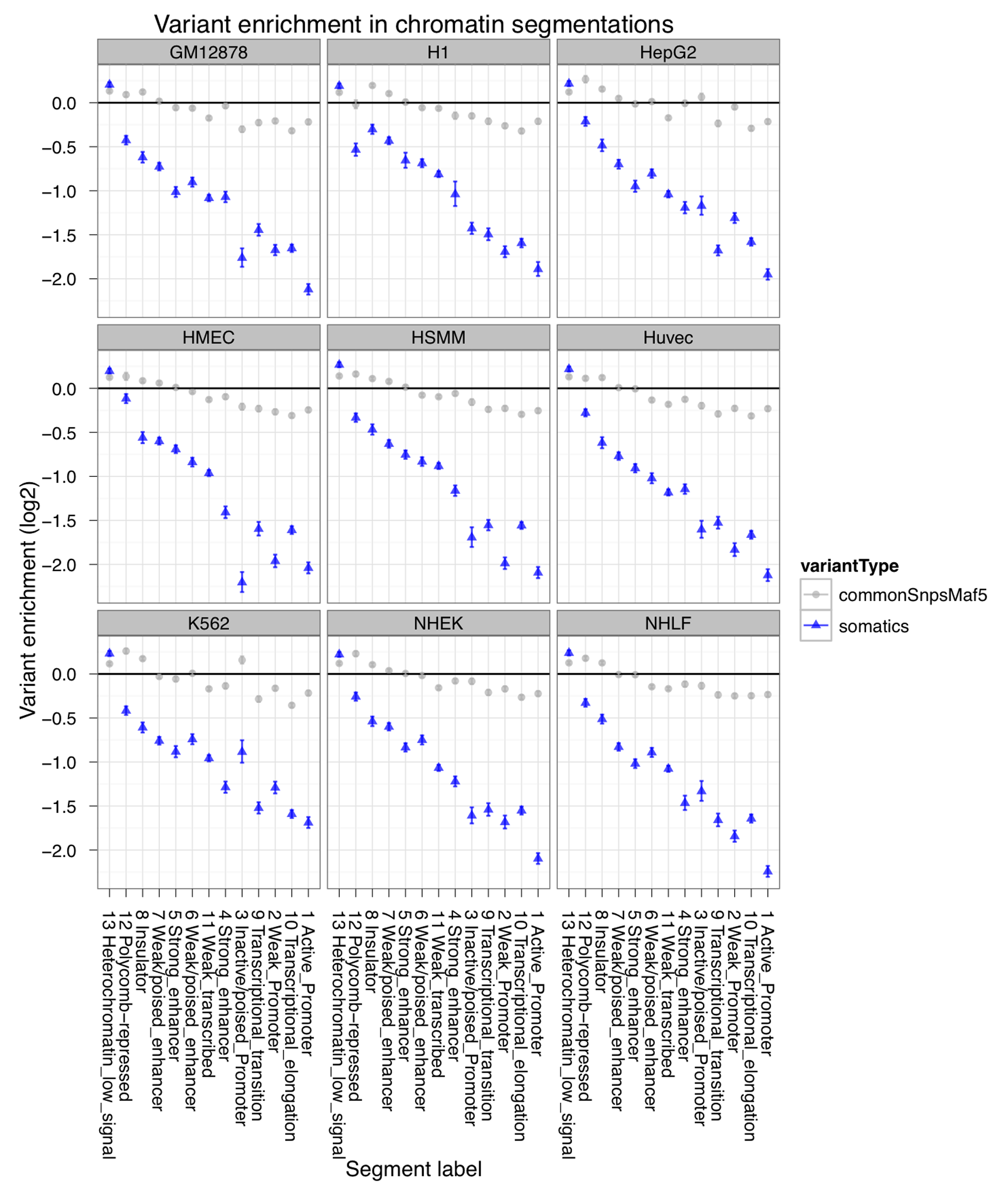

Supplement: Figure S10 — Variant enrichment in chromatin segmentations across nine different cell types. (TIF) [file pgen.1002871.s010.tif]

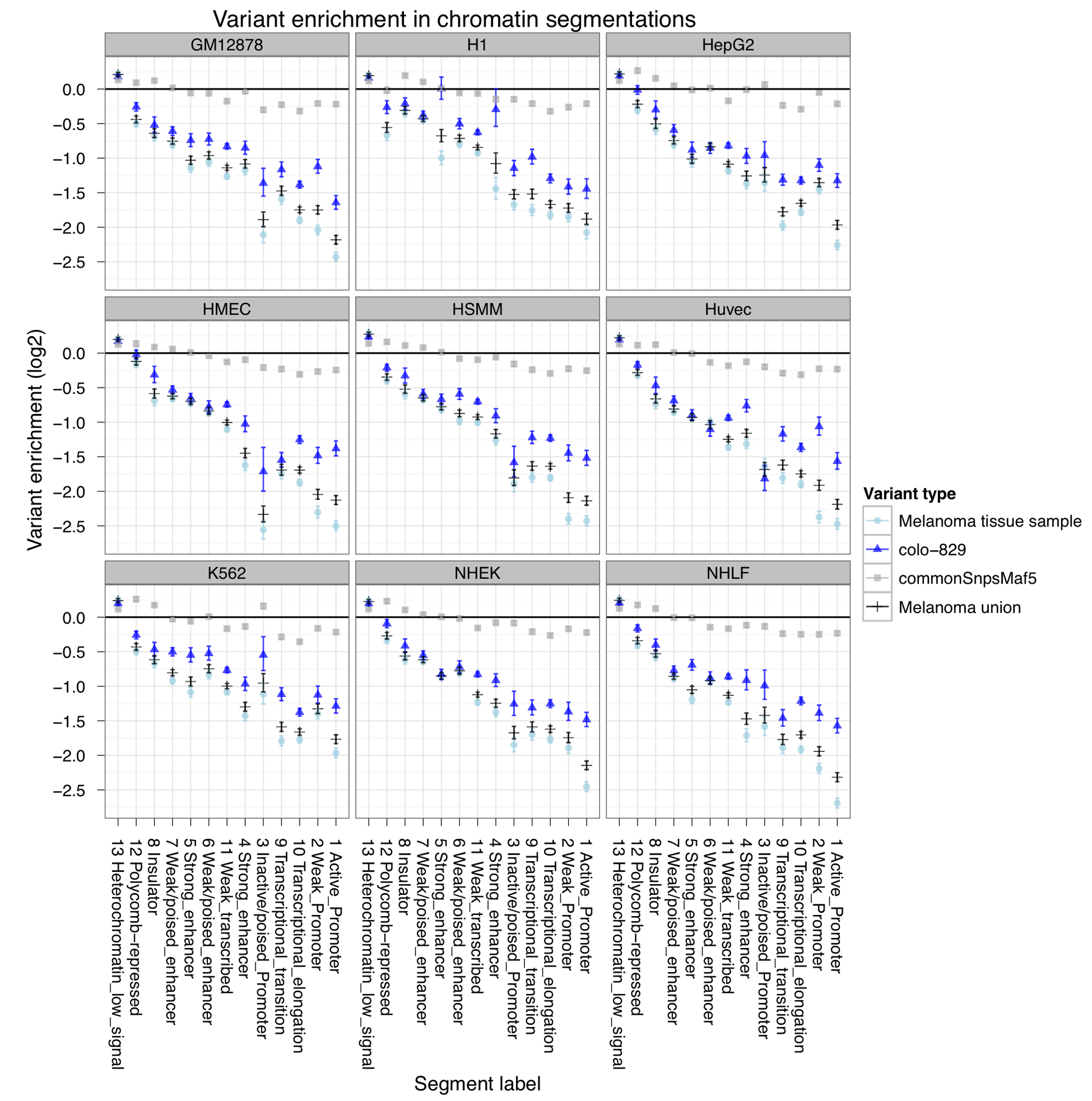

Supplement: Figure S11 — Variant enrichment in chromatin segmentations across nine different cell types using samples analyzed independently. (TIF) [file pgen.1002871.s011.tif]

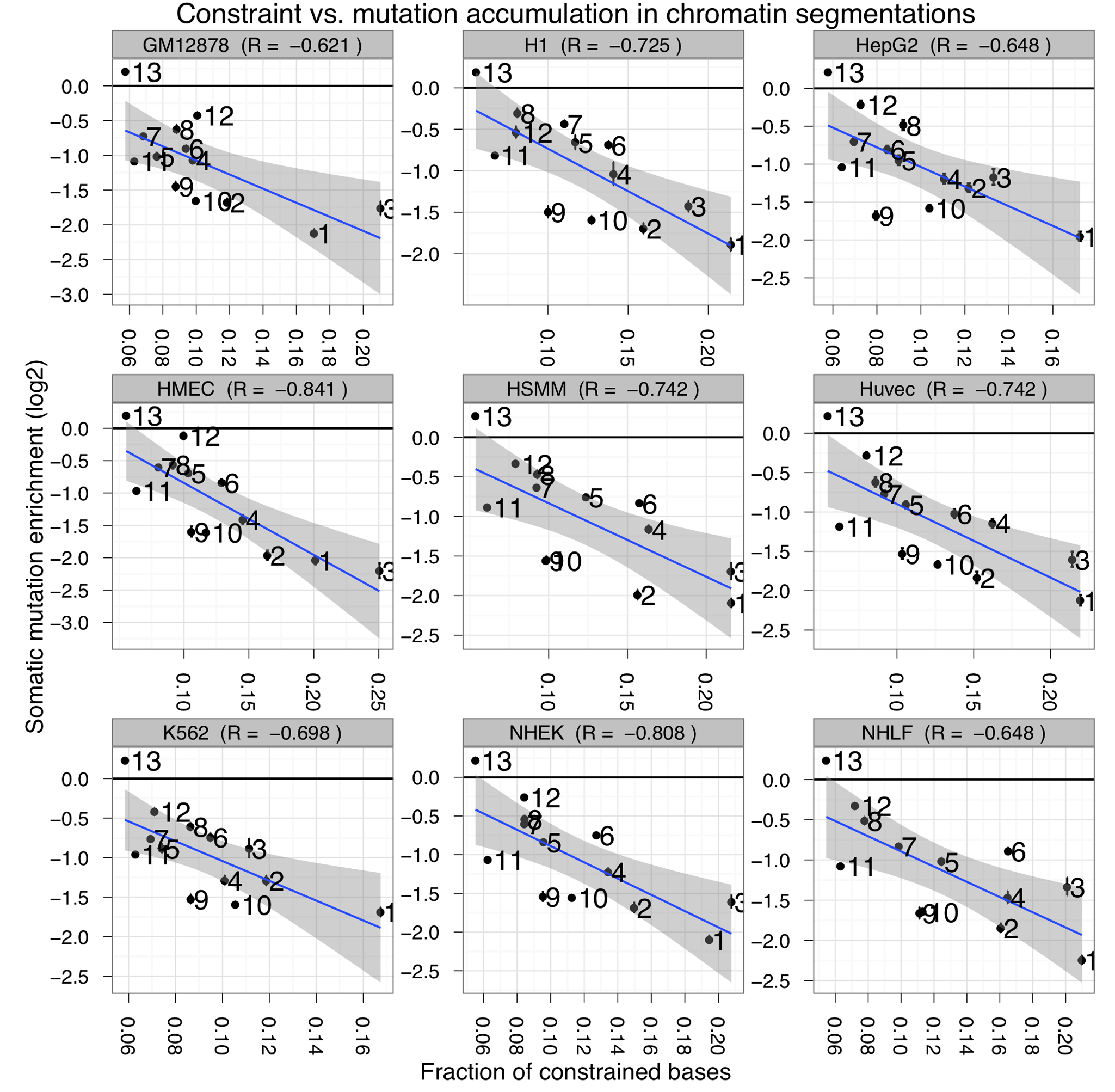

Supplement: Figure S12 — Somatic mutation enrichment compared to fraction of evolutionarily constrained bases in chromatin segmentations across nine different cell types. R values represent Spearman's correlation. (TIF) [file pgen.1002871.s012.tif]

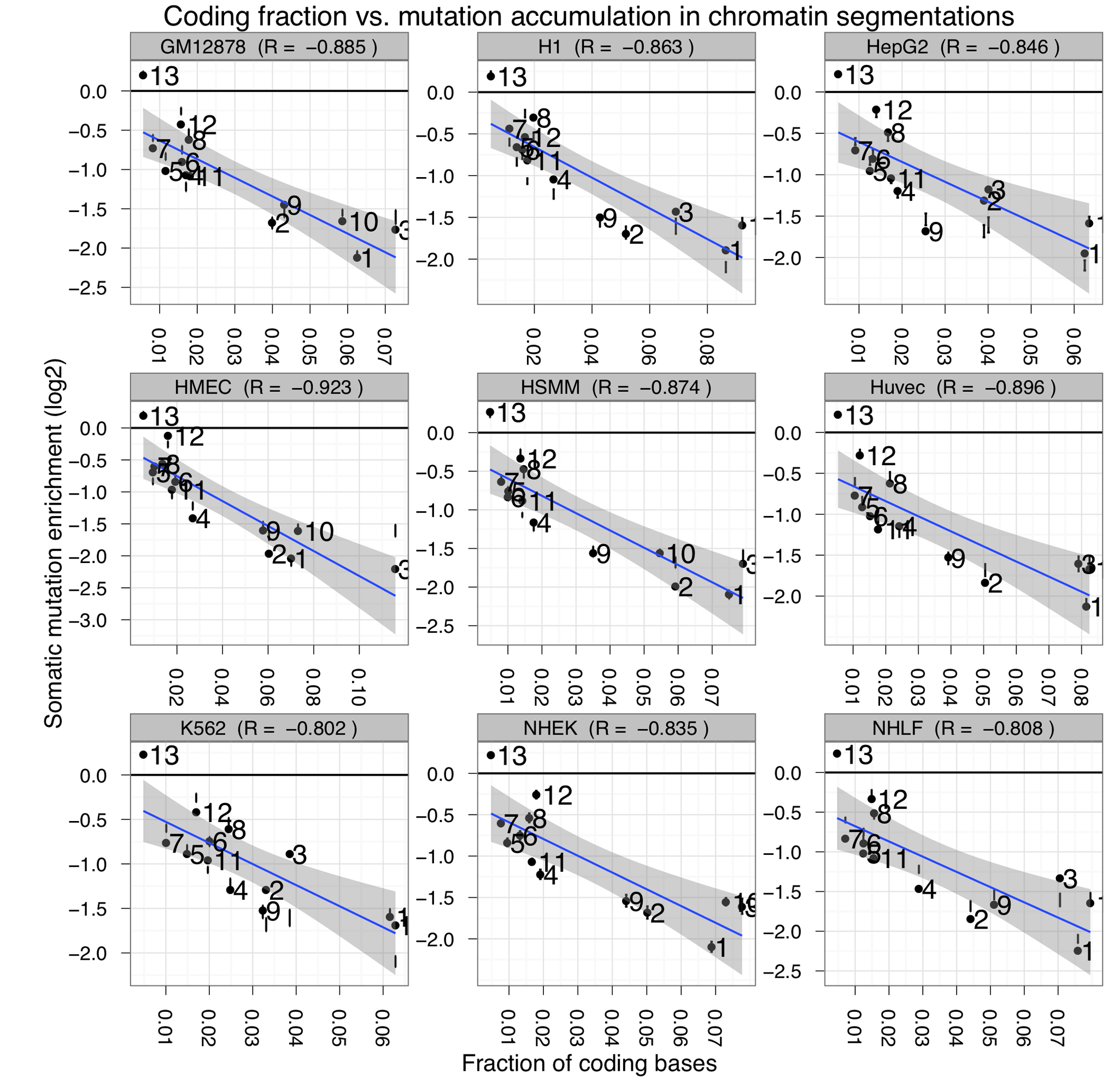

Supplement: Figure S13 — Somatic mutation enrichment compared to fraction of coding bases in chromatin segmentations across nine different cell types. R values represent Spearman's correlation. (TIF) [file pgen.1002871.s013.tif]

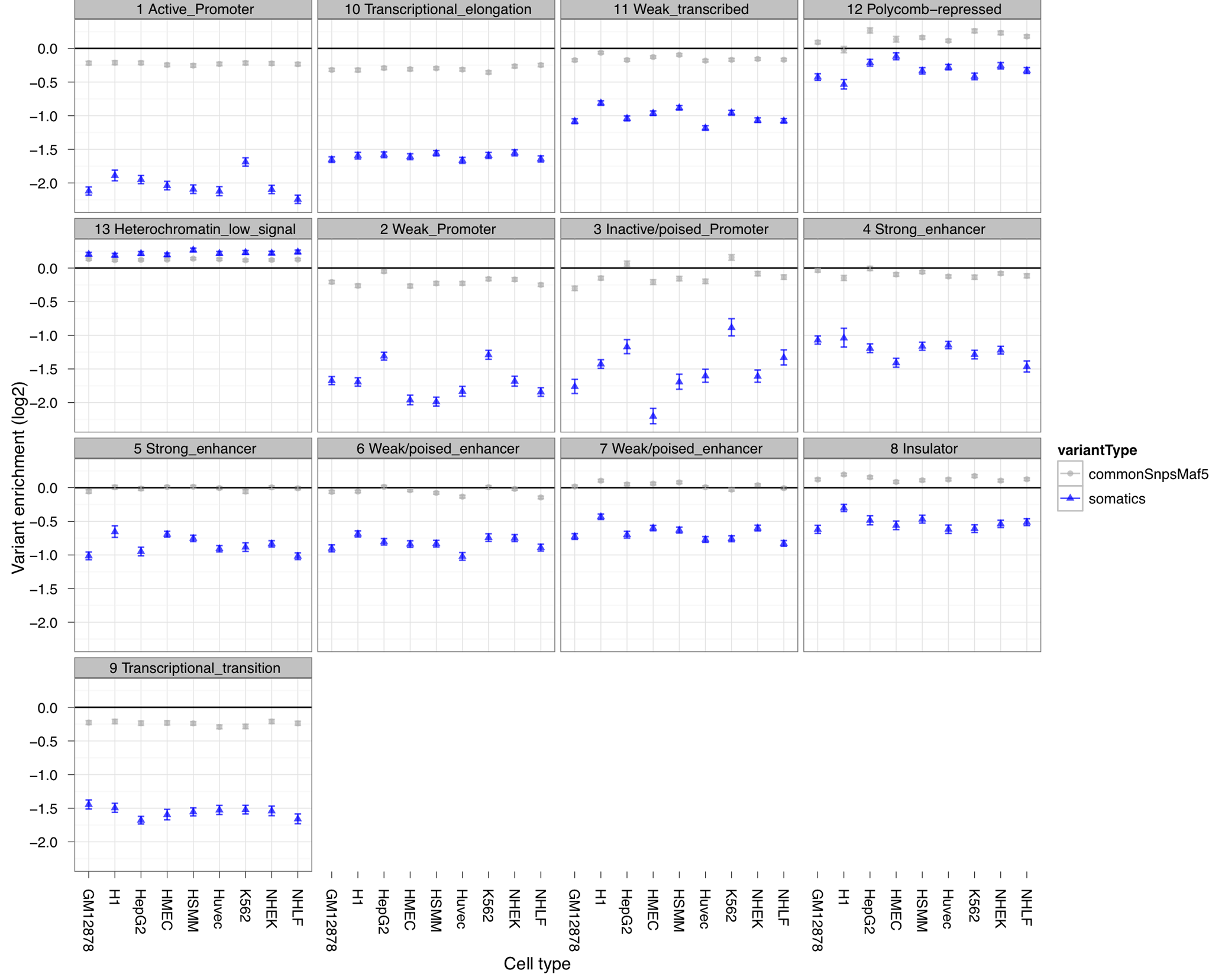

Supplement: Figure S14 — Regions that are heterochromatin low signal zones (state 13) accumulate somatic mutations at a rate similar to random expectation. (TIF) [file pgen.1002871.s014.tif]

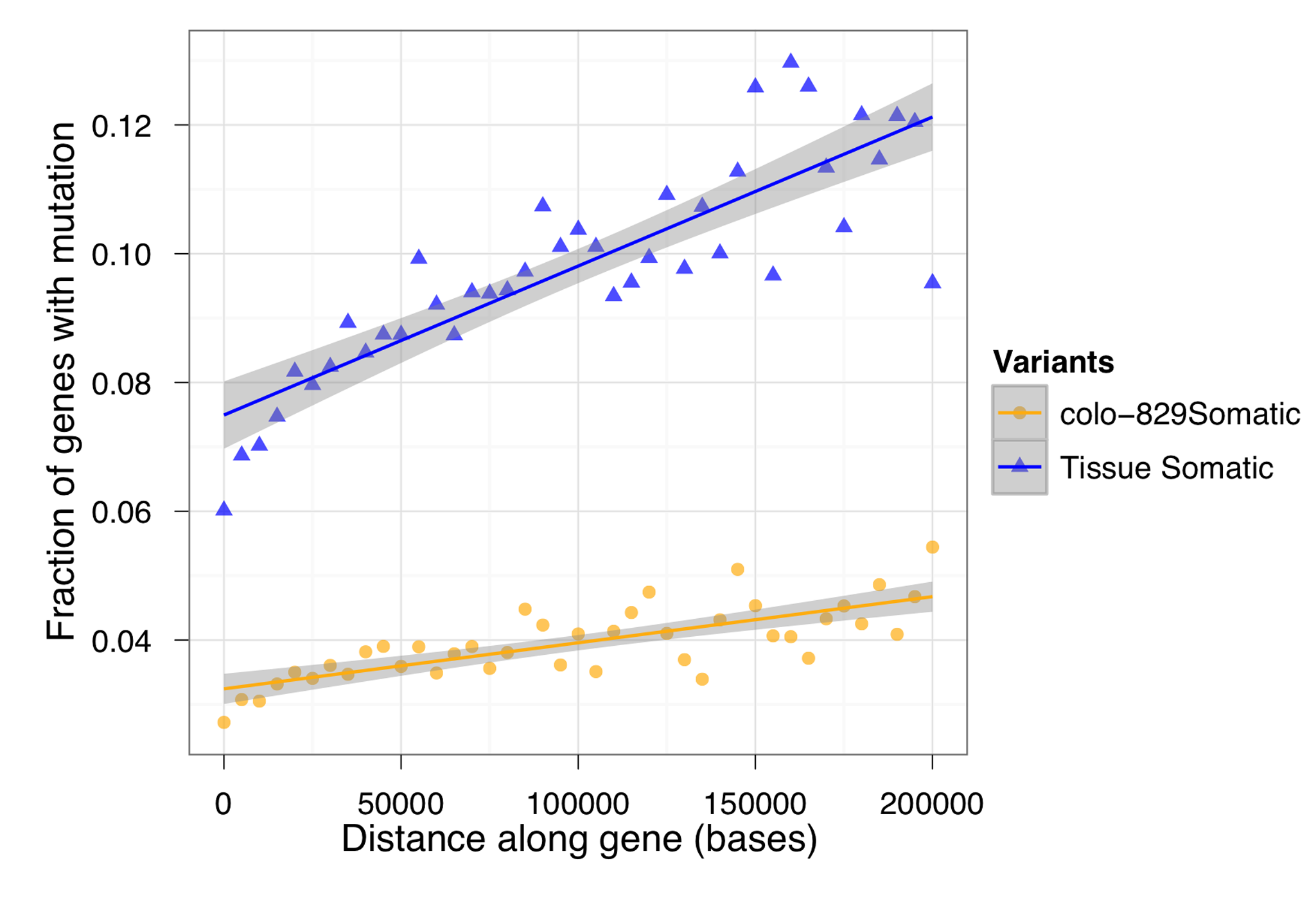

Supplement: Figure S15 — Mutation accumulation increases with distance along known transcripts. Each point represents a 5 Kb bin. (TIF) [file pgen.1002871.s015.tif]

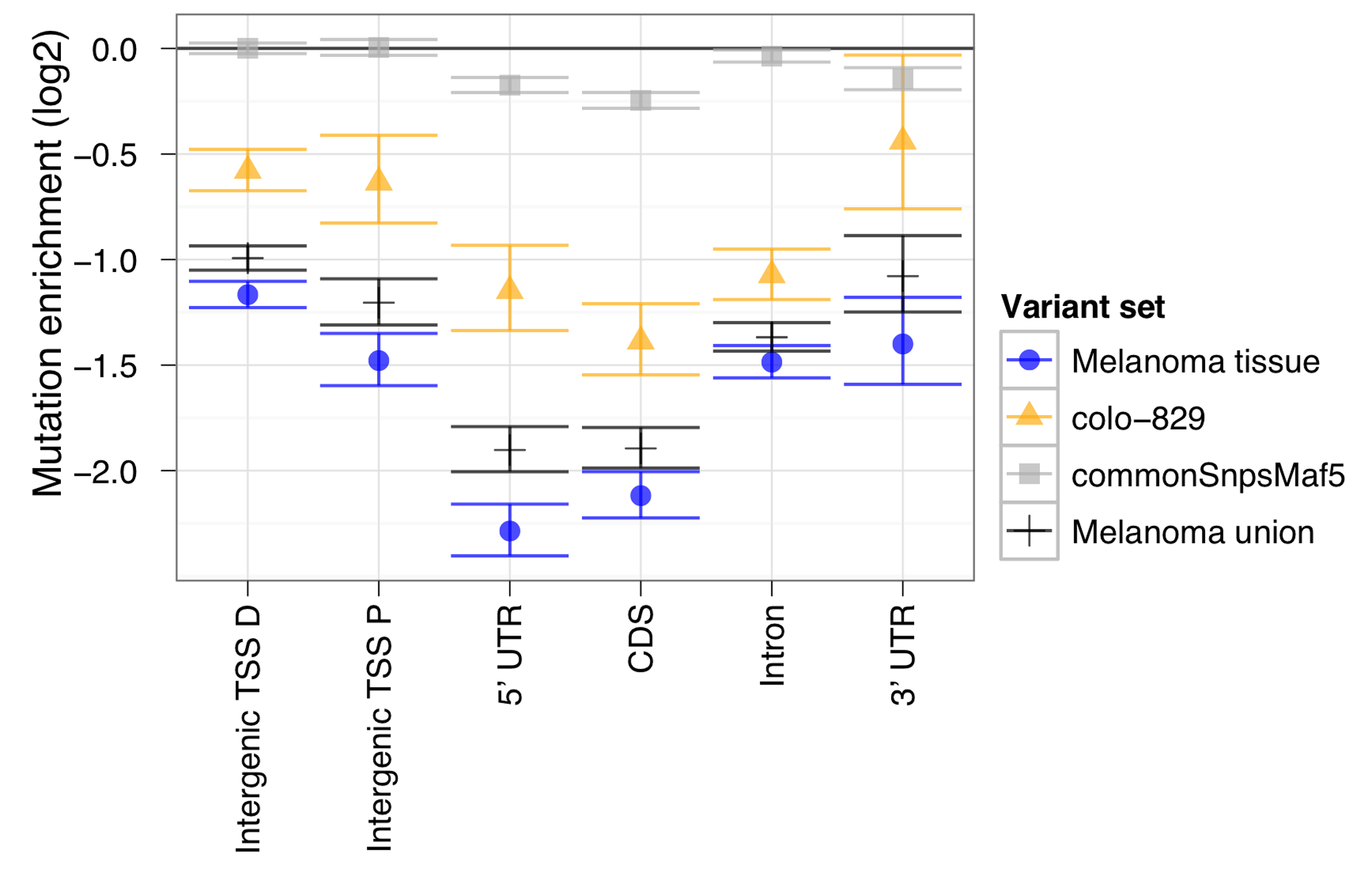

Supplement: Figure S16 — Genic partitioning of melanocyte DHSs such that every DHS occurs in a single category shows that most categories are depleted for mutation accumulation (TSS P = Transcription Start Site Proximal [within 5 Kb]; TSS D = Transcription Start Site Distal [greater than 5 Kb]). Common SNPs are based on 1000 Genomes calls that have at least 5% minor allele frequency (MAF). In addition to a union analysis, each sample is also analyzed independently in this plot. (TIF) [file pgen.1002871.s016.tif]

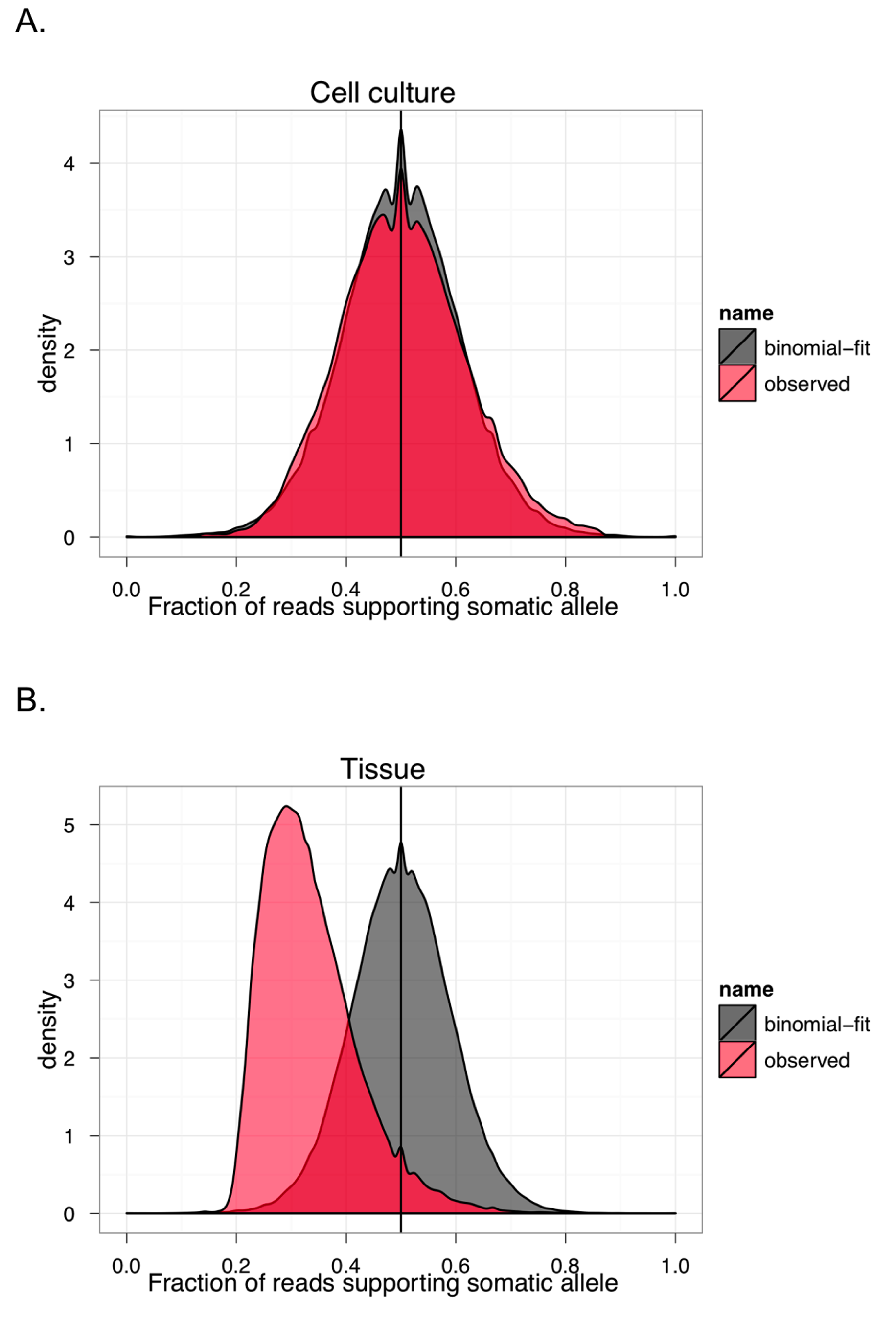

Supplement: Figure S17 — Normal cell contamination levels are different in the cell culture (A) and tissue (B) samples. We measured the fraction of MapQ30 reads that support the somatic allele at heterozygous positions and compared this to a binomial distribution fitted to the observed read counts. As expected, the cell culture has no normal cell contamination, but the tissue sample does. Based on location of the observed tissue peak at 0.29 relative to the expected peak at 0.5, we estimate the tissue sample contains approximately 42% ((0.5–0.29)*2*100) normal cells. (TIF) [file pgen.1002871.s017.tif]
